# Mixed fungal strains challenge host resistance: insights into *Magnaporthiopsis maydis* pathogenicity in maize

**DOI:** 10.3389/fmicb.2025.1520237

**Published:** 2025-01-27

**Authors:** Galia Shofman, Ofir Degani

**Affiliations:** ^1^Department of Plant Sciences, Migal – Galilee Research Institute, Kiryat Shmona, Israel; ^2^Faculty of Sciences, Tel-Hai University, Upper Galilee, Tel-Hai, Israel

**Keywords:** *Cephalosporium maydis*, crop protection, disease control, fungus, intra-species interactions, *Harpophora maydis*, late wilt, pathogenicity

## Abstract

Maize late wilt disease, caused by the fungus *Magnaporthiopsis maydis*, poses a significant threat to susceptible crops. Despite efforts to control it through resistant maize varieties, virulent fungal strains might overcome immunity. This study assessed Israeli *M. maydis* strains with weak, moderate, and highly pathogenic degrees in two open-air pot trials. Even weak pathogenic strains harmed susceptible cultivars (17% growth suppression and 33% death). In contrast, resistant cultivars were minimally affected, except when exposed to a highly aggressive isolate, resulting in a 5% growth suppression and 11% mortality at harvest. Unexpectedly, in a susceptible cultivar during sprouting, a mixed inoculum with the two more virulent isolates resulted in reduced disease (15%) compared to the highly aggressive strain alone (33%). At harvest (day 84), this pattern was reversed, and adding a weak virulent strain to this combination led to more severe growth (33%) and health (71%) disruption, accompanied by a higher level of *M. maydis* infection (371% compared to the aggressive strain alone). Similar interactions were found in other strain groups tested. Additionally, some subspecies groups specialize in growth suppression, while others in wilting, suggesting biotrophic/necrotrophic variations. The study revealed complex interactions in mixed populations, emphasizing the destructive potential of the pathogen to resistant cultivars. Understanding the role of maize age-related immunity in disease generation uncovers risks associated with this pathogen.

## 1 Introduction

Over the years, plant diseases associated with fungi have increased geographical distribution, causing more reported instances, augmented virulence, and a wider range of hosts (Fisher et al., 2012). Many factors, including environmental conditions, agricultural practices, previous infections, and the susceptibility of different plant varieties to diseases, influence the spread and outcome of these diseases (Mueller et al., 2020). With the continuous growth of the global population, it becomes increasingly important to boost agricultural output to ensure sufficient food supply. However, the heavy use of fertilizers and chemical pesticides harms the environment and poses potential health risks. Therefore, reducing their use is necessary while maintaining crop yields (Alabouvette et al., 2009). Developing effective strategies for protecting crops and safeguarding the environment is an ongoing scientific effort as part of the global attempt to promote a sustainable approach to agriculture.

Maize (corn, *Zea mays*) is the leading world grain crop (Zhao et al., 2017; Tanklevska et al., 2020), together with rice and wheat (Food and Agriculture Organization of the United Nations, 2022). It is considered a staple food for humans and fodder for animals. In addition, it has applications in various industries, including producing food additives, oils, starch, paper, and biofuel (Tanklevska et al., 2020). However, the full yield potential of maize crops is often not realized due to a range of diseases. These diseases not only reduce the quantity and quality of the maize kernels but also increase the costs associated with growing the crop (Subedi, 2015; Munkvold and White, 2016; Mueller et al., 2020). In regions like the Mediterranean Sea and South Asia, the hot and humid climate fosters the development of these diseases, and economic limitations prevent the development of effective prevention methods, causing ongoing challenges in maize production (Khokhar et al., 2014).

Late wilt disease (LWD), caused by the fungus *Magnaporthiopsis maydis* [synonyms include *Cephalosporium maydis* and *Harpophora maydis* (Samra et al., 1963; Gams, 2000; Klaubauf et al., 2014)], is typical for such climate zones and considerably impacts maize production in Egypt, Israel, Spain, Portugal, India, and other permissive climate regions (so far, it has been reported in 10 countries, Degani, 2021; Matos et al., 2024). Symptoms of late wilt are often not readily observable in the early stages of the plant’s life (Sunitha et al., 2020). These symptoms become apparent once the pathogen invades and occupies the vascular system of a vulnerable plant, obstructing its water conduction (typically starts near flowering, 50 days from sowing onwards) (Drori et al., 2013; Ortiz-Bustos et al., 2019). This blockade can be attributed to mycelium, spores, or polysaccharides produced by the fungus (Sabet et al., 1970b; Mahatma and Mahatma, 2015). The wilting phenomenon might also arise from damage to the root system (Molinero-Ruiz et al., 2010; Tej et al., 2018). The wilt disease manifests as relatively rapid and comprehensive plant dehydration. The symptoms include color loss in leaves and other green components of the plant and damage to the cobs (if seeds are produced, they are defectively developed). Severe cases (heavily infected areas planted with susceptible maize hybrid) can potentially lead to significant yield loss (Degani and Cernica, 2014; El-Naggarr et al., 2015; Degani et al., 2019b). The pathogen association with other plant pathogenic fungi like *Fusarium verticillioides*, the causal agent behind stalk rot, and *Macrophomina phaseolina*, the charcoal rot disease agent (all grouped in the post-flowering stalk rot disease complex), can enhance the damage to the maize plants (Khokhar et al., 2014; Subedi et al., 2016; Degani et al., 2020a; Elsayed et al., 2023). On the other hand, members of the seed’s microbiome can counteract the pathogen, thereby reducing symptoms (Degani et al., 2021a; Degani et al., 2024a.).

The inherent persistence of the fungus in the soil (Sabet et al., 1970a), on maize stubble (Sabet et al., 1970b; Singh and Siradhana, 1987b), and alternative host plants (Sabet et al., 1966; Sahab et al., 1985; Dor and Degani, 2019) obstruct efforts to control the pathogen effectively. The prevalent LWD control relies on an agrotechnical approach to form a suite of strategies grounded in avoidance, exclusion, and eradication principles (Degani, 2022). Utilizing resistant hybrid varieties that exhibit substantial defense against LWD is central to this approach (Degani et al., 2022b; Rakesh et al., 2022; Abdelghany et al., 2023). Leveraging the resistance of particular cultivars is seen as an efficient, economical, and eco-friendly solution, often favored over chemical and biological interventions. Yet, continuing development and field examination of new hybrids are required since LWD immunity could be compromised. Since the 1980s, the introduction of genotypes exhibiting resistance has substantially mitigated the economic repercussions associated with the disease in Egypt (El-Shafey et al., 1988) and Israel (Degani and Gordani, 2022; Degani et al., 2022b). Such breeding programs for developing resistant hybrids operate in India as well (Gazala et al., 2021). Among the commercial varieties of maize, a spectrum of LWD sensitivities exists (Kamara et al., 2021; Sunitha et al., 2021). Absolute resistance to late wilt disease is uncommon, as is a complete absence of disease symptoms; nevertheless, utilizing varieties exhibiting moderate to high resistance is common even though their market demand is often lower than susceptible ones. It should be emphasized that resistance is influenced not only by the maize variety but also by the pathogen’s virulence (García-Carneros et al., 2011). Indeed, the pathogen can spread in non-symptomatic resistant hybrids and infect their seeds (Drori et al., 2013; Degani et al., 2020b). Also, there is a variation in the virulence among different strains of *M. maydis*, influencing the infectivity across diverse maize cultivars (Zeller et al., 2002; Ortiz-Bustos et al., 2015; Shofman et al., 2022). Moreover, pathogens originating from distinct geographic regions demonstrate differences in their virulence and the disease severity they induce (García-Carneros et al., 2011; Ortiz-Bustos et al., 2015).

So far, researchers have studied the Egyptian pathogen isolates (Zeller et al., 2000; Zeller et al., 2002), which have engaged with a narrow assortment of *M. maydis* strains and their co-interactions. These pathogen subspecies were characterized and grouped into four distinct clonal lineages, displaying variability in virulence and colonization efficacy on maize and competitive interactions (Zeller et al., 2002). The lineage exhibiting the highest virulence (when tested in isolation) was found to be the least competitive on susceptible maize when assessed in a mixed inoculum comprising all four fungal lineages. Conversely, a lineage with lower virulence was observed to dominate 70% of infections, indicating higher competitive ability. Consequently, it was postulated (Zeller et al., 2002) that the highly virulent strain may possess a competitive advantage in the presence of a resistant maize cultivar. However, in regions where susceptible maize hybrids are prevalent, the less virulent *M. maydis* strains could become more predominant, while the most virulent lineage may be comparatively scarce. This pattern could explain why the continuous cultivation of a single resistant genotype over several years might lead to the emergence of more virulent or aggressive *M. maydis* strains (El-Assiuty et al., 1988; Ortiz-Bustos et al., 2015) capable of infecting that specific cultivar. This phenomenon was observed in Israel with the relatively resistant maize cultivar., Royalty, which became the predominant sweet maize cultivar during the late wilt disease outbreak in the 1990s (Degani et al., 2014; Degani et al., 2019a).

To enhance the breeding program to develop maize varieties resistant to the disease, it is essential, firstly, to deepen the understanding of *M. maydis*’ pathogenic diversity and the constraints posed by the fungus. The research of Zeller and colleagues (Zeller et al., 2002) has involved a limited four identified subgroups, a scope that perhaps fails to encompass the full range of variations, pathogenic potential, and host specificity. In Spain (García-Carneros et al., 2011), isolates from different regions were examined to assess the severity of the disease they induced. All the isolates triggered pronounced disease symptoms and wilting compared to the control group. While there were variations in the disease symptoms throughout the plant’s growth, the most substantial differences originated from the isolates alone, irrespective of the maize variety. In Israel, the *M. maydis* isolates are differentiated by their growth rate and pathogenicity (Shofman et al., 2022). Virulence strains are scattered throughout the country in mixed populations. Some fungal strains mainly affect plant growth, while others primarily cause disease symptoms. Moreover, different host cultivars evoke specific *M. maydis* strains’ virulence.

These findings have economic implications. Israel’s sweet corn cultivation encompasses in 2022 ca. 3,220 ha, producing an estimated 50,018 tons annually (Food and Agriculture Organization of the United Nations, 2022). Israel (together with Egypt) is considered one of the world’s most challenged areas by LWD (Degani, 2021). This outcome is likely because this region is the source of the disease (Samra et al., 1962) and also due to optimal climate conditions for its development (Magarey et al., 2011). While addressing the challenges LWD poses to maize growers demands a comprehensive approach encompassing many aspects (Degani, 2022), this Mediterranean region is ideal for testing them.

This research examines the relationship between Israeli *M. maydis* strains of varying virulence levels and their effects, individually and in combination, on two commercial cultivars with differing susceptibility. These findings aim to support risk assessment and the development of control strategies to mitigate the disease. To achieve this, two full-season, open-enclosure pot trials were conducted. The first trial assessed the impact of strain virulence on cultivar immunity, focusing on whether highly virulent strains could damage the LWD-resistant cultivar and to what extent. The second trial evaluated the combined effects of fungal strains with varying aggressiveness, collected from different regions, on plant growth and LWD symptom development. Data were collected at two critical growth stages: the end of the sprouting phase (before flowering and initial disease symptoms) and at harvest. A highly sensitive real-time PCR technique was used to detect pathogen DNA within plant tissues.

## 2 Materials and methods

### 2.1 Rationale and research design

**Figure 1:**
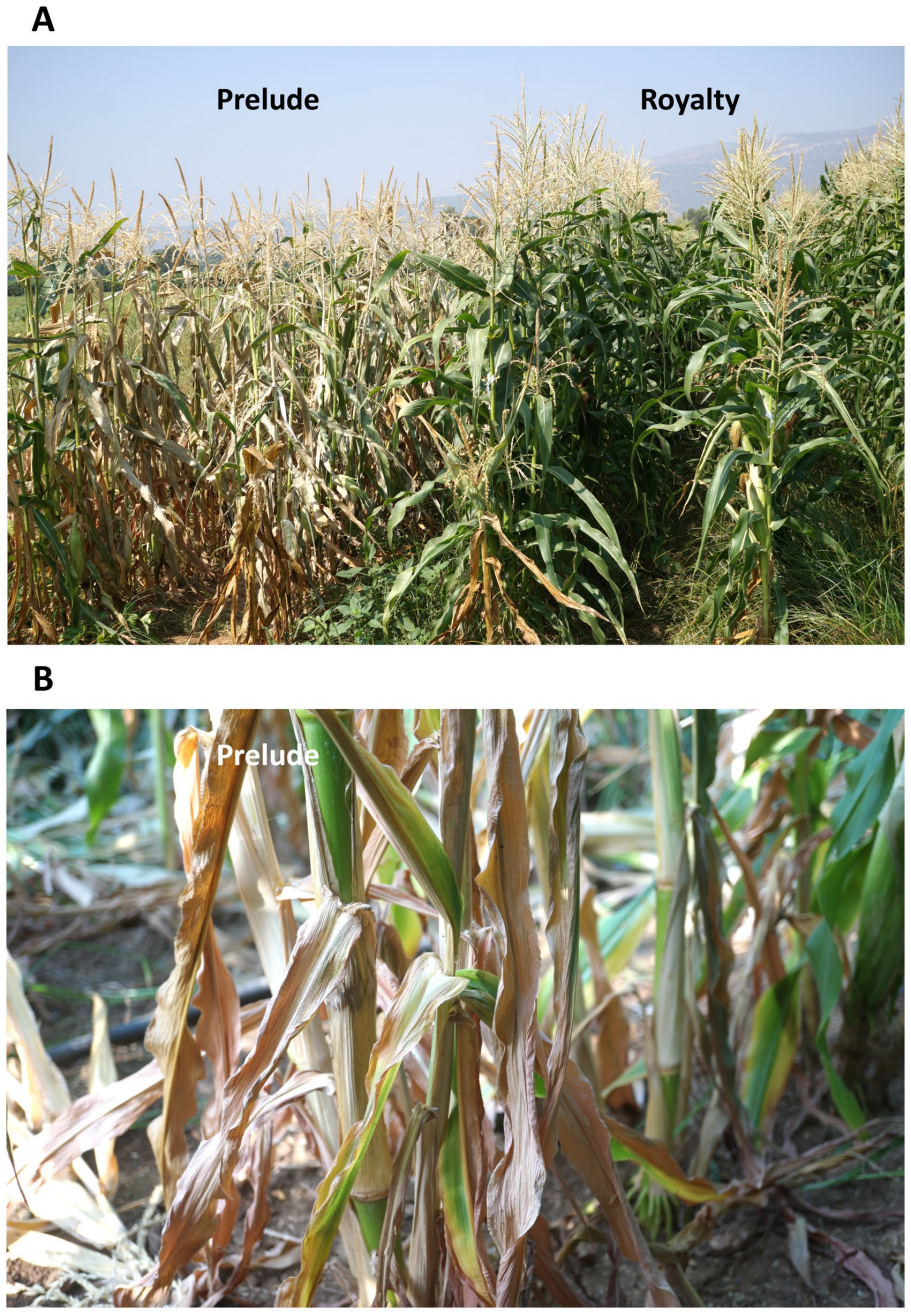
Prelude and Royalty sweet maize cultivars in the field. Photos were taken in the summer of 2016 when the plant reached 71 days from sowing. The field was used to observe and identify maize cultivars’ resistance to late wilt disease (conducted by North Israel R&D, Israel Ministry of Agriculture; Degani et al., 2019a). This area, located in the southern region of a maize field (Mehogi 5 maize plot) near Kibbutz Amir in the Hula Valley (Shemesh field crops partnership, Upper Galilee, Northern Israel), has been infested with late wilt for many years. **(A)** General overview of the two cultivars. **(B)** Closeup photo of the Prelude genotype showing severe late wilt disease symptoms.

The study was performed in two parallel experiments. The first experiment involved two maize cultivars, Prelude, a late wilt disease (LWD) susceptible cultivar, and Royalty, a LWD resistant cultivar (Degani et al., 2020b; Figure 1). Both Prelude and Royalty corn cultivars are sweet maize hybrids with similar characteristics and growth (including fertilization and harvest dates). They share close values of biomass, height, and yields (Supplementary Table S1).

Those two genotypes were challenged by three fungal strains that differ in their virulence degree (tested earlier in Shofman et al., 2022), especially to see if the most aggressive *M. maydis* isolate can compromise the immunity of the resistant cultivar.

The second experiment tested these strains, alone or in combinations, to identify cross-influence (antagonism or synergism) in inducing LWD. In addition, we assessed the influence of local isolate groups from the Upper and Lower Galilee in the north and Yavne in Israel’s coastal plain to study the impact of the geographical origin on the pathogenic traits and co-influence on the host plant. Since the *M. maydis* strains are present in the soil in mixed populations, a better understanding of their co-influence can provide a vital starting point for developing a control strategy, especially those based on antagonistic microorganisms. Such friendly bacteria and fungi can react differently against specific fungal strains or groups of strains.

Tests were conducted in pots in a semi-field (open enclosure) environment during full-season growth. Using pots in such experiments provides some advantages over field trials—a more uniform environmental setting and better control of soil composition and fertilizers, inoculation load, and irrigation management. Such a method also prevents cross-influence between treatments (while scattering them in a complete randomized design) and enables the addition of a negative control—non-infected healthy plants.

### 2.2 Fungi origin used in this study

All *M. maydis* isolates were obtained from diseased maize plants collected from commercial fields in Israel (Table 1). These strains have been previously characterized through pathogenic and physiological assessment, colony morphology, microscopic properties, and molecular identity confirmation (Drori et al., 2013; Degani and Goldblat, 2014; Degani et al., 2019a; Shofman et al., 2022). The pathogen’s colony morphological and microscopic features closely resembled strains reported in Egypt and India (Samra et al., 1963; Payak et al., 1970). The degree of *M. maydis* isolates aggressiveness was previously assessed in net house full-season potted plants, as detailed in Table 1. A reference mid-aggressive isolate designated *Mm2* (*Hm-2*, CBS 133,165) was chosen for this study. This strain is currently stored at the CBS-KNAW Fungal Biodiversity Center in Utrecht, The Netherlands, along with two other *M. maydis* strains (Drori et al., 2013): *Hm-1* (CBS 133,164) and *Hm-4* (CBS 133,166), which were not included in this work.

**Table 1:** *Magnaporthiopsis maydis* isolates used in this research.^1^

Isolate designation	Maize cultivar source	Maize type	Seed company	Isolation location (field)	Maize plants growth stage and health	Isolation date	Virulence degree^1^	References
*Mm 2* (CBS 133,165)	Jubilee	Sweet	Pop Vriend Seeds B.V., Andijk, The Netherlands	Sde Nehemia, upper Galilee, northern Israel	Near harvest, diseased	08/2001	8	Drori et al. (2013) ^2^
*Mm 16*	Leonor	Fodder	Villemur sur Tarn, France	Beit Keshet, Lower Galilee, northern Israel	Near harvest, diseased	08/2017	4	Shofman et al. (2022)
*Mm 18*	Sentinel	Sweet	HM Clause, Inc., Davis, CA, USA	Beit Keshet	Near harvest, diseased	10/2017	9	Shofman et al. (2022)
*Mm 25*	L.G.30.669	Fodder	Limagrain	Yavne, southern coastal plain	Near harvest, diseased	08/2019	12	Shofman et al. (2022)
*Mm 26*	L.G.30.669	Fodder	Limagrain	Yavne	Near harvest, diseased	08/2019	7	Shofman et al. (2022)
*Mm 27*	L.G.30.669	Fodder	Limagrain	Yavne	Near harvest, diseased	08/2019	10	Shofman et al. (2022)
*Mm 28*	32D99	Fodder	DuPont Pioneer, Johnston, IA, USA	Malkia, Upper Galilee, northern Israel	Post-harvest, diseased	09/2019	5	Shofman et al. (2022)
*Mm 29*	32D99	Fodder	DuPont Pioneer	Malkia	Post-harvest, diseased	09/2019	1	Shofman et al. (2022)
*Mm 30*	32D99	Fodder	DuPont Pioneer	Malkia	Post-harvest, diseased	09/2019	11	Shofman et al. (2022)
*Mm 32*	32D99	Fodder	DuPont Pioneer	Malkia	Post-harvest, healthy	09/2019	15	Shofman et al. (2022)

1 Based on infection experiments conducted in net house full-season potted plants, *M. maydis* isolates were previously ranked (Shofman et al., 2022) according to their degree of virulence, from the lowest to the highest among the 16 isolates tested.

2 Isolate Mm 2 was used here also as a reference strain.

Virulence tests for *M. maydis* strains were conducted previously with increasing levels of complexity to assess their ability to cause disease symptoms (Shofman et al., 2022). The trials began with *in vitro* laboratory experiments, evaluating the impact of secreted metabolites on seed germination. This was followed by pathogenicity tests on sprouts under controlled growth room conditions for up to 20 days. Finally, a full-season experiment was conducted in a semi-field environment within a net house.

The Mm29 (weak aggressive strain) and Mm2 (moderate virulent strain) were Isolated from wilting plants in upper Galilee in north Israel. In contrast, the source of Mm25 (highly aggressive fungal strain) was from Yavne in the southern coastal plain (Shofman et al., 2022). Those three strains were studied alone, in different combinations, and altogether and compared to three additional groups of isolates: Mm16 and Mm18 (representative of the Beit Keshet, Lower Galilee, northern Israel region), Mm25, Mm26 and Mm27 (representative of the Yavne, southern coastal plain), and Mm28, Mm30 and Mm 32 (representative of the Malkia, Upper Galilee, northern Israel area).

### 2.3 Growth of the fungi

All colonies were grown on a PDA medium (Potato Dextrose Agar Difco, Detroit, MI, United States) in 90 mm plates. The plates were kept in an incubator at a temperature of 28 ± 1°C in the dark for 4–5 days. To transfer the fungal colony to a fresh growth plate, a 6 mm diameter disk, obtained from the perimeter of a 5–7-day-old colony, was removed and placed in the center of a new PDA medium and incubated at the above conditions. For submerged cultures, 10 fungal disks were sown in an Erlenmeyer flask containing 150-ml potato dextrose broth (PDB; Difco Laboratories, Detroit, MI, United States). The flasks were plugged with a breathable cover and incubated for 6 days, shaken in the dark at 150 rpm at 28 ± 1°C.

### 2.4 Virulence trials in a semi-field, open enclosure, full-season pots

#### 2.4.1 Inoculation method

Soil inoculation was performed before, during, and after seeding. First, the soil was inoculated 14 days before sowing by placing six fungal colony discs (6 mm) in the designated seed planting hole. Those mycelium discs were cut from the margins of fresh pathogen colonies (5–7 days old). According to the treatments, all six discs were from the same *M. maydis* strain, three discs from each of two isolates, or two discs from three strains. Second, six mycelium discs were placed alongside each seed at sowing (Degani and Gordani, 2022). Lastly, stab inoculation using infected wooden toothpicks was performed 21 days post-sowing. The wooden toothpick inoculation method to enhance the disease was formerly tested and approved in the field (Degani et al., 2021b).

To prepare the infected toothpicks, *M. maydis* mycelium was prepared for each isolate separately by growing it in a liquid medium (PDB) for 21 days. The cultures were homogenized in a blender for 2 min, resulting in a mixture of spores and truncated mycelial fragments. The combined homogenized cultures of different fungal strains were used when such combinations were applied. Toothpicks were autoclave sterilized and subsequently incorporated into the isolates’ mixture 24 h before their utilization in the plants’ inoculation. Control group toothpicks were soaked in sterile PDB.

This infection occurred when the seedlings had grown sufficiently to avoid serious injury from the piercing process. Every plant was stabbed near the base of the stem, as near the soil as possible, and the infected wooden toothpicks were left stuck in the stalk. Each stalk was inoculated with a single toothpick. The control maize plants were wounded with PDB-inoculated sterilized toothpicks.

#### 2.4.2 Growth conditions

The experiment’s detailed timetable is seeding—03/08/2022, thinning to one plant/pot—14/09/2022 (42 days after sowing, DAS), fertilization—28/09/2022 (56 DAS), and harvest—26/10/2022 (84 DAS). The experiment included 10 *M. maydis* strains (Table 1) applied separately or in combinations. The trial was conducted in 10-L pots placed in an open enclosure at Avni Etan Experimental Farm located in the Golan Heights (north-eastern Israel, 32°49′03.3″ N 35°45′46.4″ E). The experiment’s local peat soil was from a field with no known history of LWD infection. This infection is assumed to be minor and lacks significant influence if found to exist. It was mixed with 20% perlite No. 4 for aerating the ground.

The experiment included 10 biological repeats (pots per treatment). Due to late wilt disease mortality, the final number of surviving repeats is indicated for each figure. Each pot was sown with five Prelude cv. or Royalty cv. seeds. The seeds were pre-treated with a mixture of thiram, captan, carboxin, and metalaxyl-M, a standard general pesticide treatment. Seed samples were tested before sowing to ensure high vitality. Computerized drip line irrigation was carried out using 2 liters per pot daily. According to the plants’ needs, adjustments in the water routine and pesticide handling to prevent other diseases from influencing the plants were conducted during the growth (corresponding to the Ministry of Agriculture’s recommended growing protocol). During the maize cultivation season, the average temperature was 24.5°C, with a minimum of 12.6°C and a maximum of 40.8°C. The average humidity was 66.1%, with a minimum of 18.0% and a maximum of 95.7%. Such conditions permit disease outbreaks (Singh and Siradhana, 1987a), but the temperatures were below the optimal of 26–28°C (Degani et al., 2021b). Still, the intense inoculation pressure applied here allows disease development.

#### 2.4.3 Assessment of plants’ growth and health

The metrics used for the growth and health assessments were according to the standard parameters adopted in previous experiments (Gordani et al., 2023). The growth parameters of the plants were recorded on the thinning day and at the experiment ending: survival percentage, plant height, fresh weight of above-ground parts, phenological stage (leaves’ number), and flower count. Also, root samples were taken and freeze-stored for future DNA extraction and purification. At the end of the semi-field open encloser trial (day 84), additional parameters were evaluated: ear weight and number and late wilt symptom assessment. The evaluation of wilt signs within various plant organs, such as the stalk base (first above-ground internode), leaves, cob husks, and the plant as a whole, was categorized into four groups: dead, severe (showing clearly noticeable signs of wilting), mild (displaying minor signs of dehydration), and healthy (Degani et al., 2021b).

### 2.5 Real-time PCR *M. maydis* DNA analysis

A quantitative real-time PCR (qPCR) detection was applied to track *M. maydis* DNA levels in the plant tissues (roots up to day 42 and first above-ground stem internode at day 84) (Degani and Gordani, 2022). DNA was extracted from the tissues of 8–10 plants per treatment (unless otherwise indicated). The plants’ parts were washed thoroughly with running tap water, then incubated in 1% sodium hypochlorite (NaOCl) and sterile tap water for 10 min each. The plants’ parts were cut into ca. 2 cm sections, and the total weight of each repetition was set to 0.7 g.

The plant and fungal total DNA was isolated and extracted according to a previously published protocol (Murray and Thompson, 1980) with slight modifications (Degani et al., 2019a). Briefly, 0.7 g of maize plant tissue was ground with 4 mL of cetyl hexadecyltrimethylammonium bromide (CTAB). 1.2 mL was transferred to the microfuge test tubes and heated at 65°C for 20 min. The tubes were centrifuged at 13,000 rpm at room temperature (24°C) for 5 min, and the supernatant (700 μL) was moved to new tubes with an equal volume of chloroform/isoamyl-alcohol (24:1). The last cleaning stage was repeated twice. The upper segment (300 μL) was transferred to a new microfuge test tube with 200 μL of previously cooled isopropanol, and the test tubes were maintained at 20°C for 20–60 min. The DNA was then concentrated at 12,000 rpm for 20 min at 4°C, and the liquid was removed. The DNA precipitate was redissolved with 0.5 mL of ethanol. After an additional concentration and ethanol wash cycle, the DNA precipitate within the tubes was desiccated in a sterile environment overnight. Subsequently, the DNA was dissolved in 100 μL of ultra-pure water and maintained at −20°C until utilized for qPCR.

The molecular detection method is based on a standard qPCR protocol (Livak and Schmittgen, 2001) optimized to detect *M. maydis* DNA using species-specific primers (Zeller et al., 2000; Saleh and Leslie, 2004). The A200 primers are explicitly designed for *M. maydis*, amplifying a segment initially identified as specific through amplified fragment length polymorphism (AFLP) (Saleh and Leslie, 2004). The mitochondria’s last enzyme in the cellular respiratory electron transport chain, the cytochrome C oxidase COX gene, served as a housekeeping reference to normalize the relative amount of *M. maydis* DNA in the samples examined (Garrido et al., 2009; Almquist, 2016; Baskarathevan et al., 2016). The COX gene was amplified using the COX F/R primer set (Weller et al., 2000; Li et al., 2006). The relative gene abundance was calculated according to the ΔCt model, and the same efficacy was assumed for all samples. The primer pair was tested previously (Degani et al., 2019b) and proved suitable for application to maize. The qPCR method was verified (the curves validated that the appropriate products were amplified and quantitated).

All qPCR reactions were performed in four technical repeats. Amplifications were made using the CFX 384 Real-Time PCR Detection System (Bio-Rad, Hercules, CA, United States) and 384-well plates. The volume of the reaction was 5 μL/well. Each well contained 0.25 μL of the forward and backward primers (at 10 μM concentration), 2.5 μL of iTaq™ Universal SYBR Green Supermix solution (Bio-Rad Laboratories Ltd., Hercules, CA, USA), and 2 μL of diluted DNA sample. Reaction conditions were 95°C for 60 s (pre-cycle activation phase), followed by 40 cycles of 95°C denaturation phase for 15 s, 60°C for 30 s (annealing and extension), and finally, the formation of a melting curve.

### 2.6 Statistical analysis

The semi-field potted experiments were conducted in a completely randomized statistical design. No random effect with statistical significance was found in the pots’ setting. Analysis and statistical processing were performed using GraphPad Prism software, version 10.1.0 (316) 17/10/2023 (GraphPad Software Inc., San Diego, CA, United States). The data were analyzed utilizing a one-way analysis of variance (ANOVA) and posterior Dunnett’s test (which restricted to comparing the experimental groups against a single control group) at a significance level of *p* < 0.05. Due to the difficulty of uniformly inoculating the plants, high variability in the pot assay results (high standard error values) is expected. This makes it challenging to obtain statistically significant differences. To maintain the power of the test and reduce the risk of type II errors, we opted not to adjust for multiple tests and used Fisher’s least significant difference (LSD) test.

## 3 Results

This study examined representative *Magnaporthiopsis maydis* strains with an elevated virulence degree: (1) to challenge two commercial maize hybrids with distinct late wilt disease (LWD) immunity—resistant and highly susceptible, and (2) to study their pathogenic threat either alone or in combinations in comparison to other isolates groups collected in divers region across Israel.

**Figure 2:**
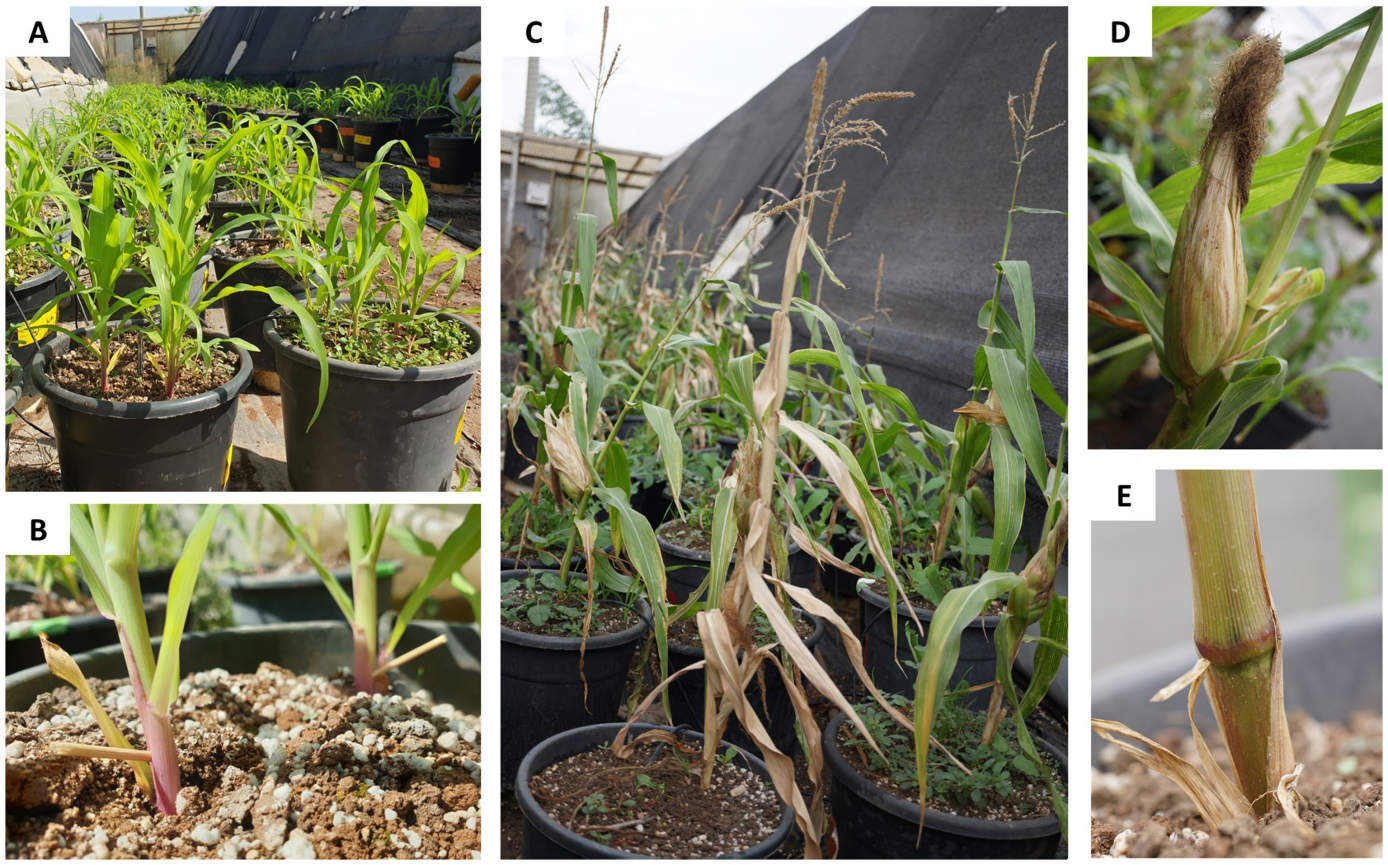
The semi-field open-enclosure full-season pots trial photos. This trial was carried out at Avni Etan Experimental Farm in the Golan Heights (north-eastern Israel) with the Prelude cv. (late wilt disease susceptible) and Royalty cv. (late wilt disease resistance). **(A)** The mid-season (day 42) experiment photo. **(B)** Toothpick stabbing inoculation at the above-ground lower portion of the stem. At the sprouting phase, the plants were vivid with no signs of disease and were not affected by the injury caused by the toothpick inoculation. **(C)** The end-season (day 84) photos. At the season-ending, many of the treatments’ plants were diseased and showed various dehydration stages. In addition to vitality and overall plant health status, the typical LWD disease symptoms were observed as an increasing number of dry leaves and symptoms appearing on the cobs husks **(D)** and in the lower part of the base of the stalk (first above-ground internode, **E**), as described earlier (Drori et al., 2013; Farahat et al., 2020).

Previous work outcomes (Shofman et al., 2022) facilitated the segregation of the *M. maydis* isolates into three virulence groups, from which representative isolates (Table 1) were chosen for the current study: Mm 29 (low virulence), Mm 2 (moderate virulence), and Mm 25 (high virulence). These isolates were evaluated here over an entire growth season in open-air potted plants (Figure 2) against two maize varieties: the Royalty cv., which exhibits tolerance to LWD, and the Prelude cv., known to be susceptible to the disease (Degani et al., 2019a).

The plants were developed well without noticeable disease symptoms at the sprouting phase (day 42 post-seeding, Figure 2A). The toothpick-stabbing inoculation conducted on day 21 did not harm the plants’ development, monitored 21 days later (Figure 2B). Yet, at harvest (84 days post-sowing), some treatments led to severe LWD outbreaks (Figure 2C). The whole plant was at advanced wilting stages in the most severe cases. Other plants showed typical LWD symptoms (Drori et al., 2013; Farahat et al., 2020), including drying out ascends upwards in the plant, leaf yellowing and dehydration, increasing dehydration patches on the cobs’ husks (the outermost leafy layers that enclose and protect the maize cob, Figure 2D), and color alteration of the stalk base (near the first above-ground internode) to a red-brown (Figure 2E).

### 3.1 The impact of isolates with varied virulence on susceptible and resistant maize cultivars

**Figure 3:**
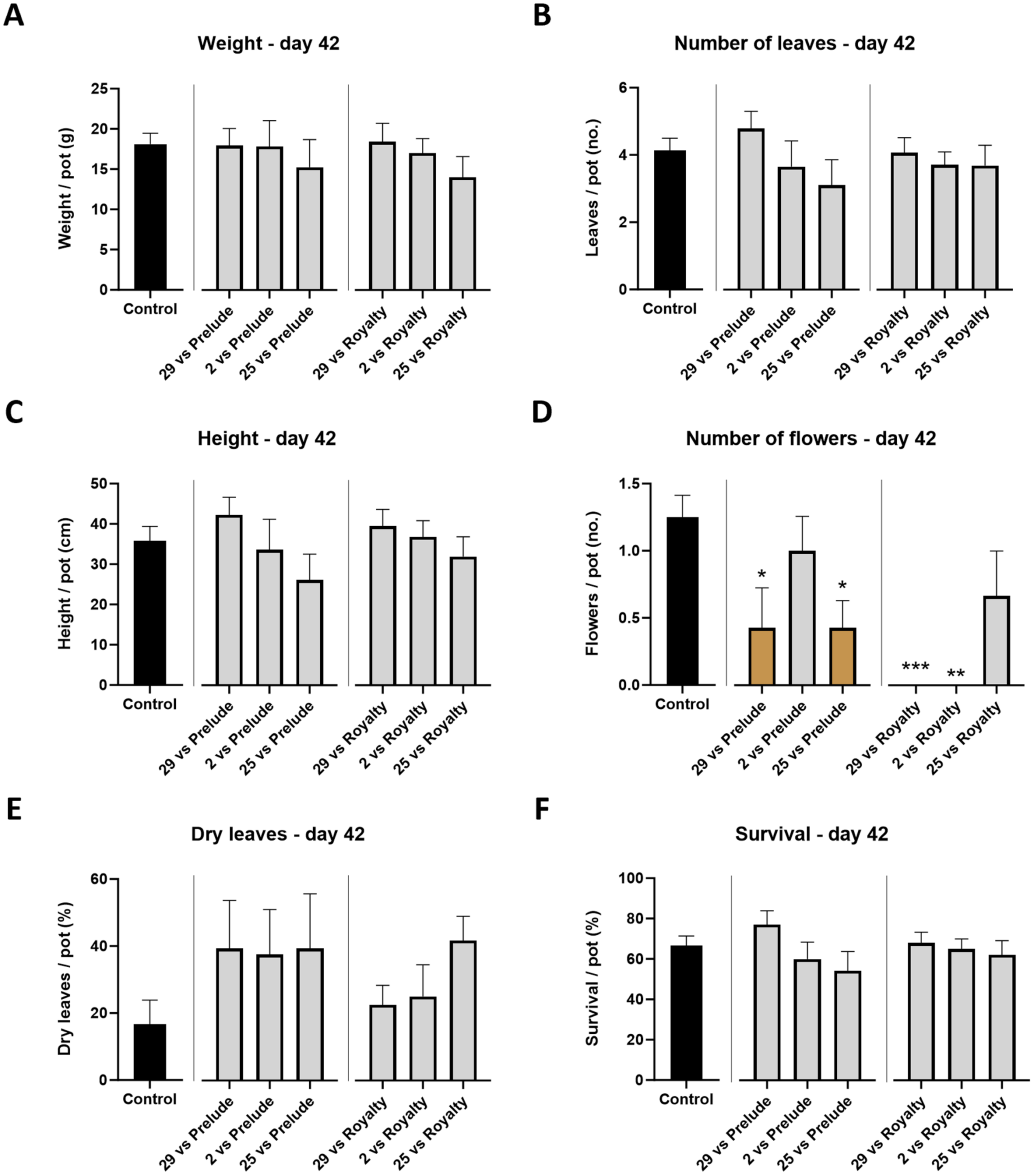
Aggressiveness toward maize hybrids—day 42. Effect of the *M. maydis* strains’ aggressiveness degree on the development and health of Prelude cv. (late wilt disease susceptible) and Royalty cv. (late wilt disease resistant) sprouts. The *M. maydis* isolates (Table 1) are representative of three aggressiveness degrees: Mm29 (29, low virulence), Mm2 (2, moderate virulence), and Mm25 (25, high virulence). **(A)** Wet biomass, **(B)** phonological stage (leaves count), **(C)** plant height, **(D)** number of flowers, **(E)** dry leaves count, and **(F)** the surviving plants’ percentage. Control— healthy uninfected Prelude cv. plants. Each value is a mean of 7–10 repetitions (average/pot per treatment). Error bars indicate the standard error. The statistically marked dissimilarity between each group and the control (highlighted in black) was tested using the one-way ANOVA assay and is represented by brown bars and different asterisks above the chart bars (*p < 0.05, **p < 0.005, ***p < 0.0005).

This part of the study examined whether *M. maydis* strains with different aggressiveness capabilities toward susceptible Prelude maize genotype exert a similar effect on the Royalty maize cultivar., known for its resistance to LWD. At the end of the sprouting phase (growth day 42), the LWD symptoms are generally expected to be latent. Indeed, mid-season sampling data were, in most cases, lacking statistically significant compared to the healthy control plants (Figure 3). Still, a constant increase in the strains’ impact on the plant’s growth and health parameters was correlated to the fungal strain virulence degree, with Mm29 as the least influential and Mm25 as the most dominant. For example, the plants’ height, leaves number, and survival rate decreased by 38, 35, and 30% in the Prelude cv., and 9, 19, and 8% in the Royalty cv. between those two strains (Mm29 and Mm35). Nonetheless, only a slight visible difference was measured between the two cultivars tested at this plant age, with better performance to the royalty genotype, as predictable. Such differences reached statistical significance in only one parameter: the flower count. The data showed drastically (*p* < 0.05) lower flowering when the plants were infected with Mm29 and Mm25 in the prelude cv., and even more with Mm29 and Mm2 in the Royalty cv. (Figure 3D). Interestingly, under Mm25 infection, the Royalty cv. plants’ flowering maintained higher (statistically equal to the uninfected control).

**Figure 4:**
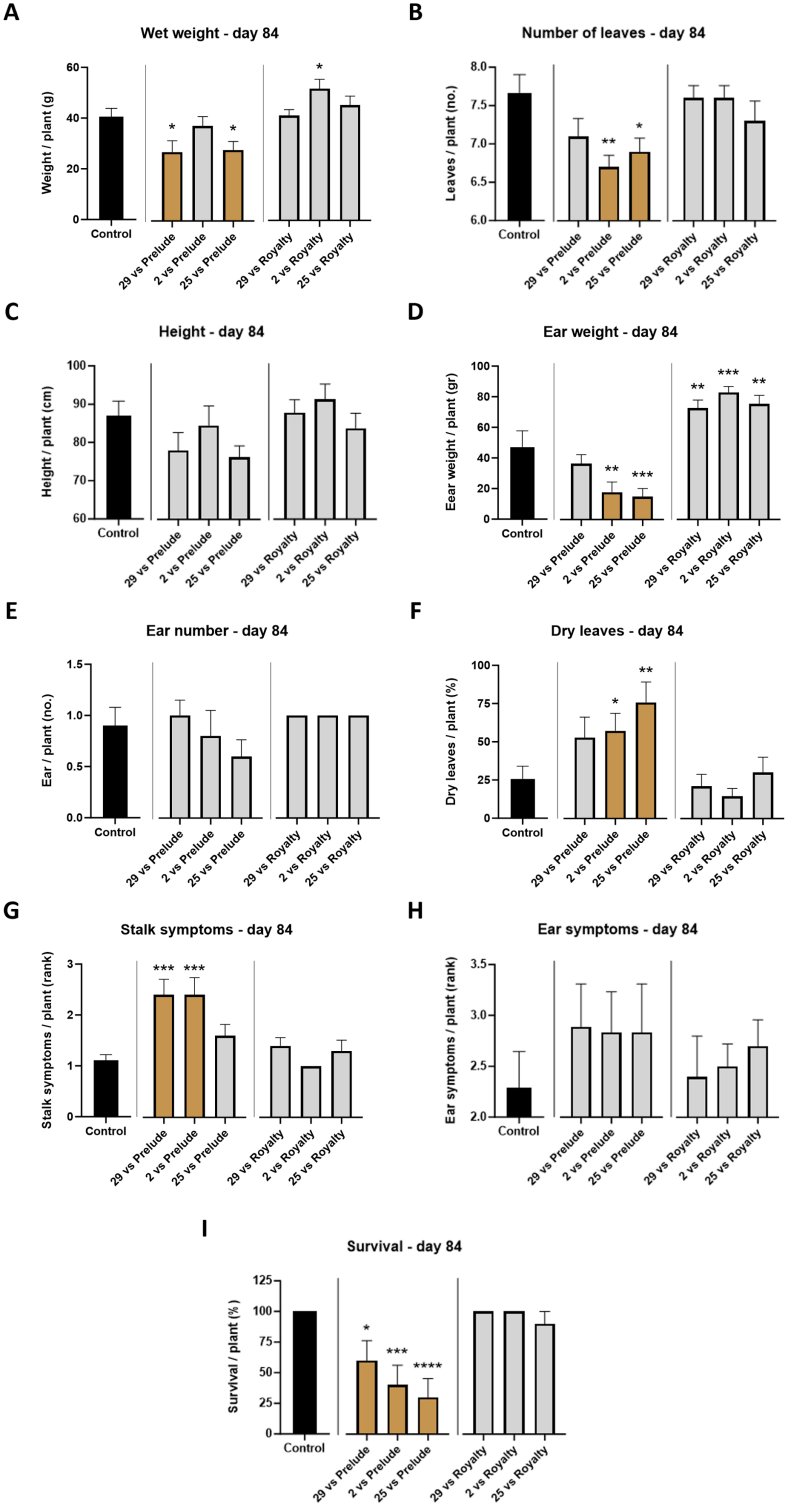
Aggressiveness toward maize hybrids—day 84. The *M. maydis* isolates (Table 1): Mm29 (29, low virulence), Mm2 (2, moderate virulence), and Mm25 (25, high virulence) were applied to challenge Prelude cv. (late wilt disease susceptible) and Royalty cv. (late wilt disease resistant). **(A)** Wet biomass, **(B)** number of leaves, **(C)** plant height, **(D)** ear weight, **(E)** ear number, **(F)** number of dry leaves, **(G)** lower stalk symptoms, **(H)** the cobs’ husks symptoms and **(I)** the surviving plants’ rate. Control—healthy uninfected Prelude cv. plants. Each value is a mean of 7–10 repetitions (average/pot per treatment). Error bars indicate the standard error. The statistically marked dissimilarity between each group and the control (highlighted in black) was tested using the one-way ANOVA assay and is represented by brown bars and different asterisks above the chart bars (*p < 0.05, **p < 0.005, ***p < 0.0005, ****p < 0.00005).

All treatments resulted in a high percentage of dry leaves (the number of dehydrated leaves relative to the total leaf count, 35 to 150% higher, compared to the control), with Mm25 emerging as the leading strain in this category. The wilting of leaves (those near the soil surface at first) is usually the first sign of LWD (Degani and Cernica, 2014) and is expected to increase over time. Indeed, in the Prelude cv., such a scenario was diagnosed on day 84, with a high percentage of dry leaves (as will be elaborated below). In contrast, the Royalty cv. had low levels of wilting leaves, like the healthy control (Figure 4).

**Figure 5:**
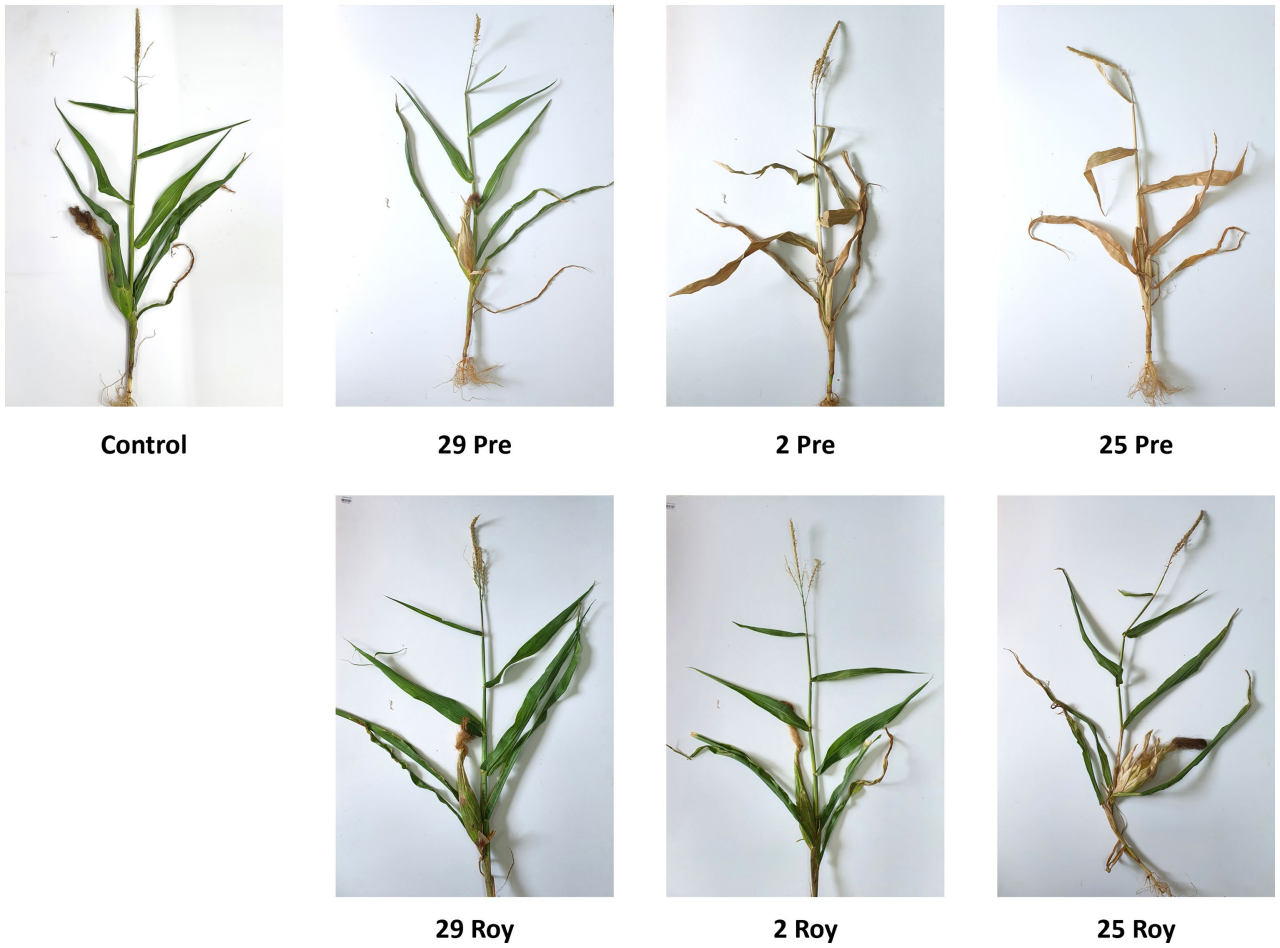
Aggressiveness toward maize hybrids (day 84)—symptoms’ photos of representative plants. The *M. maydis* isolates (Table 1): Mm29 (29, low virulence), Mm2 (2, moderate virulence), and Mm25 (25, high virulence) were applied to challenge Prelude cv. (Pre, LWD susceptible) and Royalty cv. (Roy, LWD resistant). Control—healthy uninfected Prelude cv. plant. A typical plant from each treatment was arbitrarily selected and photographed.

At the harvest (day 84 post-sowing), observations indicated that the susceptible cultivar Prelude was severely affected by all three pathogen strains, with the two more virulent (Mm2 and Mm25) being the most harmful (*p* < 0.05) regarding the ear weight, number of leaves, dry leaves, and survival rate (Figures 4, 5). The least aggressive strain, Mm29, significantly impaired the plants’ weight and caused drastic high stalk symptoms (120% compared to the mock treatment) and a low survival measure (33% mortality) in this cultivar. As anticipated, the LWD immune Royalty cv. displayed tolerance to the infection in all cases. The high virulence isolate, Mm25, inflicted the most damage against the sensitive maize cultivar but minorly impacted the resistant one. Within the resistant maize cultivar group, this aggressive isolate (Mm25) induced a 5% reduction in height and number of leaves compared to the control group. With respect to the other strains (Mm29 and Mm2), the highly pathogenic Mm25 isolate was conspicuously virulent toward the resistant hybrid, with 42–106% more dry leaves and 9–20% more ear symptoms, up to 33% more stalk symptoms and 11% higher death rate.

**Figure 6:**
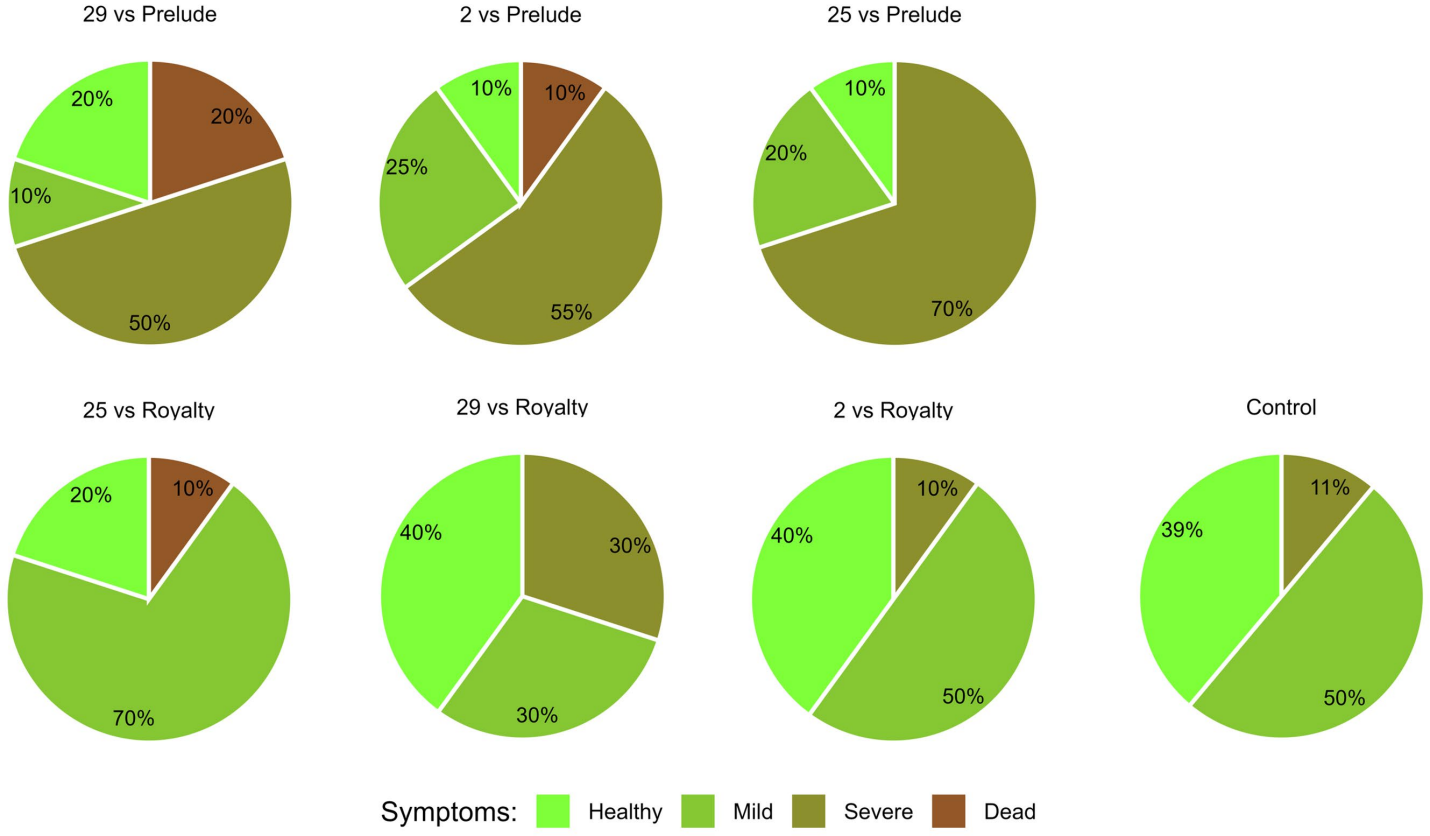
Aggressiveness toward maize hybrids day 84—comprehensive wilt disease symptoms evaluation. The *M. maydis* isolates (Table 1): Mm29 (29, low virulence), Mm2 (2, moderate virulence), and Mm25 (25, high virulence) were applied to challenge Prelude cv. (late wilt disease susceptible) and Royalty cv. (late wilt disease resistant). The overall plant health was estimated on the harvest day using four categories, as described before (Gordani et al., 2023): 1—healthy, 2—mild, 3—severe, and 4—dead.

The overall plant health estimation at the harvest (day 84) (Figure 6) aligns with the signs of wilt disease, which were documented by focusing separately on the dry leaves number and lower stems and ears symptoms. Across all parameters summarized in this four-category scale, Prelude cv. demonstrated a heightened susceptibility to the disease. Isolate Mm25 was the most aggressive in this genotype, with only 10% healthy plants. Isolate Mm2, a more moderately aggressive strain, exhibited similar vitality, with less severe disease cases but a higher mortality rate. The weakest virulent isolate (Mm29) was noticeably less harmful to the plants. When those strains tested against Royalty cv., a similar pattern was documented but with lesser LWD symptoms (20, 40, and 40% of healthy plants treated with isolates Mm25, Mm2, and Mm29, respectively).

Comparative analysis summarizing the total impact of all measures collected in both sampling days of the semi-field trial (Table 2A) highlights the different responses of the two cultivars that reflect their LWD immunity. Their tolerance was also correlated with the pathogen’s ability to cause disease. For instance, by harvest the development process was most substantially disrupted when the virulent Mm25 was introduced in both cultivars tested, causing a 39% total measures reduction in the sensitive Prelude cv. and 7% in the tolerant Royalty cv. The Prelude cv. showed an increased sensitivity to the pathogen (i.e., disease signs) with age. At the same time, the Royalty cv. was affected by the infection at the sprouting phase (day 43) but recovered and had healthy growth data at the season-ending (day 84). Among all measures, the number of green leaves was the most dominant symptom affected by the fungus at the sprouting phase, whereas the survival rate was the highest responsive index at maturation.

**Table 2:** Aggressiveness toward maize hybrids—a comparative evaluation of the growth and survival indices.^a^

(A)												
Treatment	Day 42 (average/pot)					Day 84 (average/plant)					Total (Day 84)	
	Survival (%)	Green leaves (%)	Height (cm)	Leaves (no.)	Weight (g)	Survival (%)	Green leaves (%)	Height (cm)	Leaves (no.)	Weight (g)	Ave	Rank
Control	100%	100%	100%	100%	100%	100%	100%	100%	100%	100%	100%	2
29 vs. Prelude	116%	73%	118%	116%	92%	67%	63%	90%	93%	67%	62%	5
2 vs. Prelude	90%	75%	94%	88%	98%	33%	38%	97%	87%	92%	59%	7
25 vs. Prelude	81%	73%	73%	75%	84%	22%	33%	88%	90%	69%	61%	6
29 vs. Royalty	102%	93%	110%	98%	94%	100%	106%	101%	99%	101%	97%	3
2 vs. Royalty	98%	90%	103%	90%	102%	100%	115%	105%	99%	127%	107%	1
25 vs. Royalty	93%	70%	89%	89%	77%	89%	94%	96%	95%	112%	93%	4

(B)														
Treatment	Day 42 (average/pot)				Day 84 (average/plant)				Total				All	
	Growth		Health		Growth		Health		Growth		Health			
	Ave	Rank	Ave	Rank	Ave	Rank	Ave	Rank	Ave	Rank	Ave	Rank	Ave	Rank
Control	100%	3	100%	1	100%	4	100%	2	100%	3	100%	1	100%	2
29 vs. Prelude	108%	1	94%	3	83%	6	46%	5	96%	4	70%	5	79%	5
2 vs. Prelude	94%	5	83%	5	92%	5	34%	7	93%	6	58%	7	72%	6
25 vs. Prelude	77%	7	77%	7	82%	7	46%	6	80%	7	61%	6	68%	7
29 vs. Royalty	101%	2	98%	2	101%	3	95%	3	101%	2	96%	3	98%	3
2 vs. Royalty	98%	4	94%	4	111%	1	104%	1	104%	1	99%	2	103%	1
25 vs. Royalty	85%	6	82%	6	101%	2	87%	4	93%	5	84%	4	89%	4

a The open encloser full season pots trial growth and survival indices’ summery. The *M. maydis* isolates (Table 1): Mm29 (29, low virulence), Mm2 (2, moderate virulence), and Mm25 (25, high virulence) were applied to challenge Prelude cv. (late wilt disease susceptible) and Royalty cv. (late wilt disease resistant). (A) The results were analyzed by calculating the differences in percentages of each treatment in each sampling day to the control—healthy, non-infected maize plants. The total average for all treatments’ impact on day 84 was calculated, and the treatments were ranked according to their total average (from the highest—number 1 to the lowest—number 7). (B) The summarization of growth (phenological development and above-ground parts weight and height) and health indexes (mortality and LWD symptoms) and their total co-influence.

When summarizing the effects and the growth and health indexes (mortality and LWD symptoms) separately (Table 2B), there is a correlation between the plant growth performance and its vivid measures. Still, the health indexes were more dominant at harvest, reflecting the disease outcome. Specifically, the highest impactable value was mortality. The most virulent Mm25 led to 87% (in the Prelude cv. group) and 11% (in the Royalty cv. group) fatality rate. This isolate was followed by the two other isolates—the medium (67 and 0%) and weak infectious (33 and 0% death in the two cultivars, respectively).

**Figure 7:**
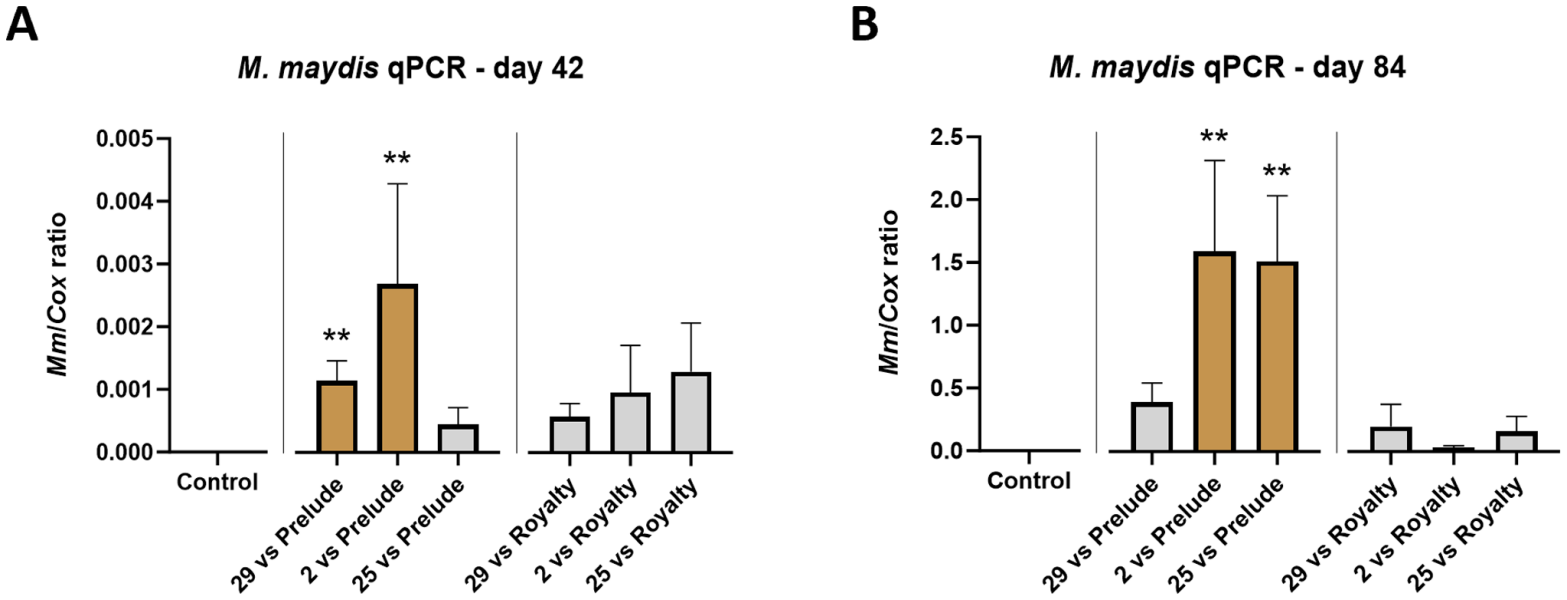
Aggressiveness toward maize hybrids—real-time PCR (qPCR) analysis. The outcome of *M. maydis* virulence on the fungal DNA amount in Prelude cv. (late wilt disease susceptible) and Royalty cv. (late wilt disease resistant) was measured in the roots (day 42, **A)** and the first above soil stalk internode (day 84, **B**). The *M. maydis* isolates (Table 1): Mm29 (29, low virulence), Mm2 (2, moderate virulence), and Mm25 (25, high virulence). The Y-axis shows the ratio of the specific *M. maydis* DNA strand to the housekeeping gene-encoding cytochrome C oxidase (COX). Values represent an average of 8–10 repetitions (plants per treatment). Error lines represent a standard error. The statistical significance of variance between each group and the control was tested using the one-way ANOVA assay and is emphasized by brown bars and different asterisks above the chart bars (**p < 0.005).

In analyzing the molecular data measured using the qPCR method, the *M. maydis* DNA quantity peaked toward the season-ending (by 337–350 fold, for example, in the Mm29 in the two maize cultivars). Also, it was notably higher (*p* < 0.05) in the sensitive Prelude maize cultivar compared to the resistant one (Royalty cv.) (Figure 7). Isolates 29 and 2 demonstrated analogous effects on both maize cultivars, agreeing with their influence on plant development and dehydration. While these responses are expected, examining the highly aggressive strain (Mm25) results reveals a less understandable picture. The DNA levels of this fungal isolate in the Prelude cv. on growth day 42 and in the Royalty cv. on day 84 were relatively low. This result is not correlated with the disease symptoms, implying that high virulence may not necessarily result from the increased presence of the fungus in plant tissues. Yet, when the Prelude cv. plants matured (day 84), The pathogen DNA increased by a factor of 3,357. This DNA increase indicates the establishment and flourishing of the pathogen colonization toward the season-ending, as documented earlier (Degani et al., 2018, 2020a). It is typical in severe cases of late wilt. The aggressive strain (Mm25) DNA elevation trend was the most significant among the three strains tested. Mm2 (the mid-aggressive strain) maintained significantly high DNA levels (*p* < 0.005) on both sampling days, and his DNA peaked by 940 fold during the plant maturation.

### 3.2 The impact of *M. maydis* isolates’ origin and intra-species interactions on late wilt disease

This research’s second part aimed to better understand the *M. maydis* intra-species interactions and the fungal stain combinations’ impact on LWD outbursts in the susceptible hybrid Prelude. The fungal strain groups were isolated from different geographical regions in Israel so that the findings could establish the link between origin and aggressiveness. To this end, the three isolates representing elevated aggressiveness degree (Mm29, Mm2, and Mm25, isolates from different locations) were combined in several variations and compared to three new isolate groups (Table 1). Those include Mm16 and Mm18 (weak virulence representative of the Beit Keshet, Lower Galilee, northern Israel region), Mm25, Mm26, and Mm27 (strains with varied virulence degree, representative of the Yavne, southern coastal plain), and Mm28, Mm30, and Mm32 (high virulence representative of the Malkia, Upper Galilee, northern Israel area).

**Figure 8:**
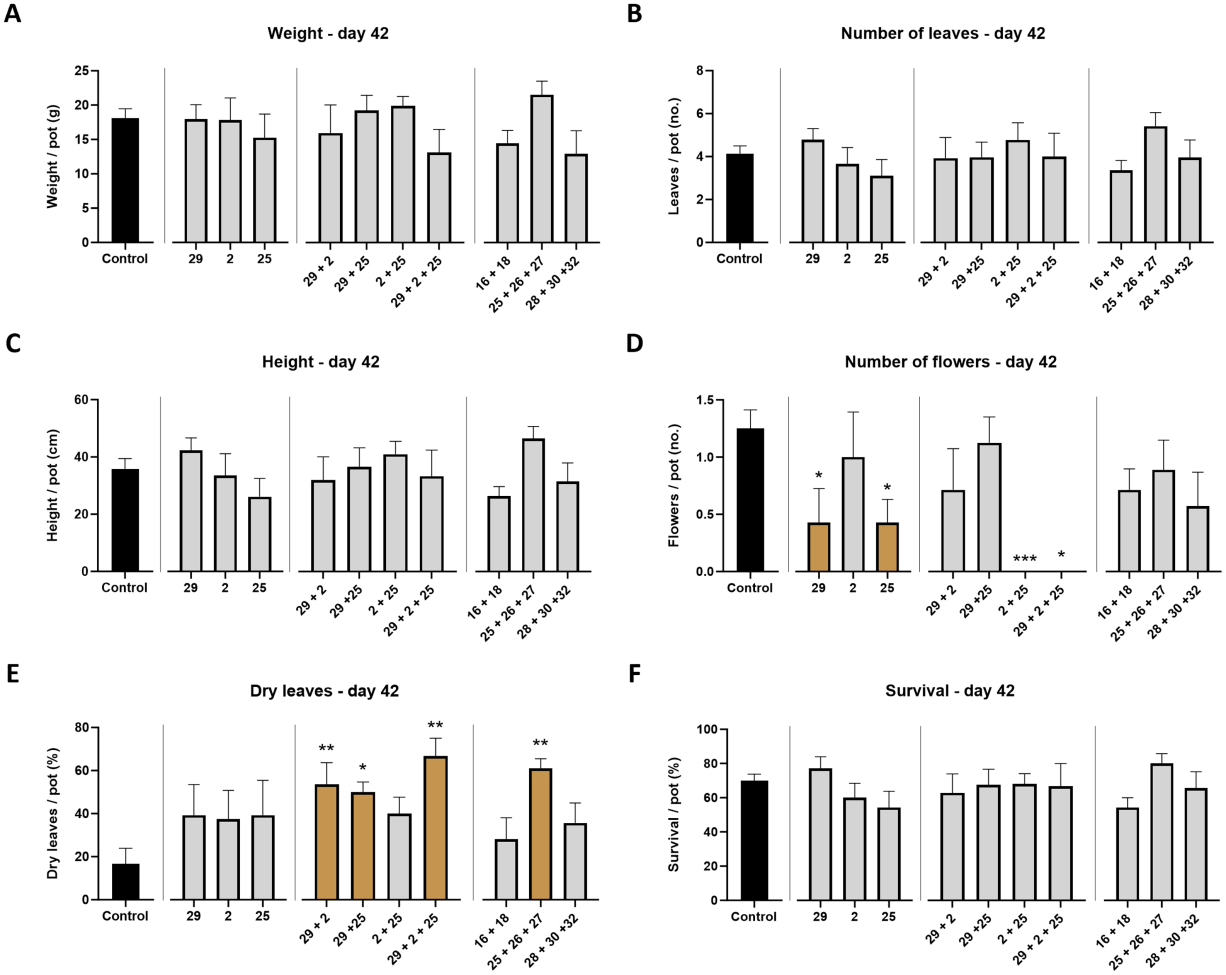
Intra-species interactions impact on maize sprouts (day 42). The effect of *M. maydis* strain mixtures on the development and health of Prelude cv. (late wilt disease susceptible) sprouts was studied. The *M. maydis* isolates (Table 1) represent different degrees of aggressiveness in Israel and its regions. These include Mm29 (29, low virulence), Mm2 (2, moderate virulence), and Mm25 (25, high virulence), Beit Keshet Mm16 and Mm18 group (16 + 18, Lower Galilee, northern Israel region), Yavne Mm25, Mm26, and Mm27 group (25 + 26 + 27, southern coastal plain), and Malkia Mm28, Mm30, and Mm32 group (28 + 30 + 32, Upper Galilee, northern Israel area). **(A)** Wet biomass, **(B)** phonological stage (leaves count), **(C)** plant height, **(D)** number of flowers, **(E)** dry leaves count, and **(F)** the surviving plants’ percentage. Control— healthy, uninfected plants. Each value is a mean of 7–10 repetitions (average/pot per treatment), except for the 29 + 2 + 25 strains group. This group includes three repeats since, on the sampling day, some pots had only one plant, and no thinning could be executed. Error bars indicate the standard error. The statistically marked dissimilarity between each group and the control (highlighted in black) was tested using the one-way ANOVA assay and is represented by brown bars and different asterisks above the chart bars (*p < 0.05, **p < 0.005, ***p < 0.0005).

Similar to the results presented above (Figure 3), on mid-season (end of the sprouting stage, day 42), the variations between the treatments were minor and, in most cases, without statistical significance (Figure 8). Nonetheless, across all measures, it is clear that the combination of the two more aggressive isolates (Mm2 and Mm 25) led to weakened disease, expressed in higher growth parameters (20–22% more height and leaves number compared to the virulent Mm25 alone), and fewer disease symptoms (18% more surviving plants relative to Mm25). This suggests antagonistic interactions between those two sub-species strains. In addition to this group that originates from different regions (Mm2 is from the north while Mm25 is from Israel’s coastal plain), the coastal plain group (Yavne’s strain group, Mm25, Mm26, and Mm27) showed similar and even intense antagonism. Their co-interactions resulted in 34% (weight), 37% (flower number), 56% (leaves count), and 57% (height) elevation in plants’ growth parameters and a 37% higher survival rate relative to Mm25 sole treatment. Still, this subspecies group could cause sharp (*p* < 0.05) dry leaves increase (131% more than in the Mm25 treatment).

The third strains combination, Mm28, Mm30, and Mm32 (representative of the Malkia, Upper Galilee, northern Israel area), was expected to cause severe LWD outbreak since it included relatively high virulent strains (ranked 5, 11, and 15 in their pathogenicity among 16 *M. maydis* subspecies, Table 1). Instead, plants infected with these strains had 13% reduced weight and 21% more dry leaves (compared to Mm25) but 11% more flowers, 21% more leaves, 15% higher plants, and a 16% better survival proportion.

In contrast, the Beit Keshet group (Lower Galilee region in northern Israel), the Mm16 and Mm18 combination, showed synergism potential. This group infection causes growth repression similar to Mm25 alone. Such a response is unexpected because those two strains are considered weak pathogenic toward maize (ranked 4 and 9 in their virulence degree, Table 1). Only the number of dry leaves was intriguingly low (67% less) compared to the Mm25 treatment (Figure 8E). In fact, the wilted leaves count was the only measure with significant values in the strain mixtures treatments, and, logically, this measure was generally opposite to the growth response (Figures 8A–C).

**Figure 9:**
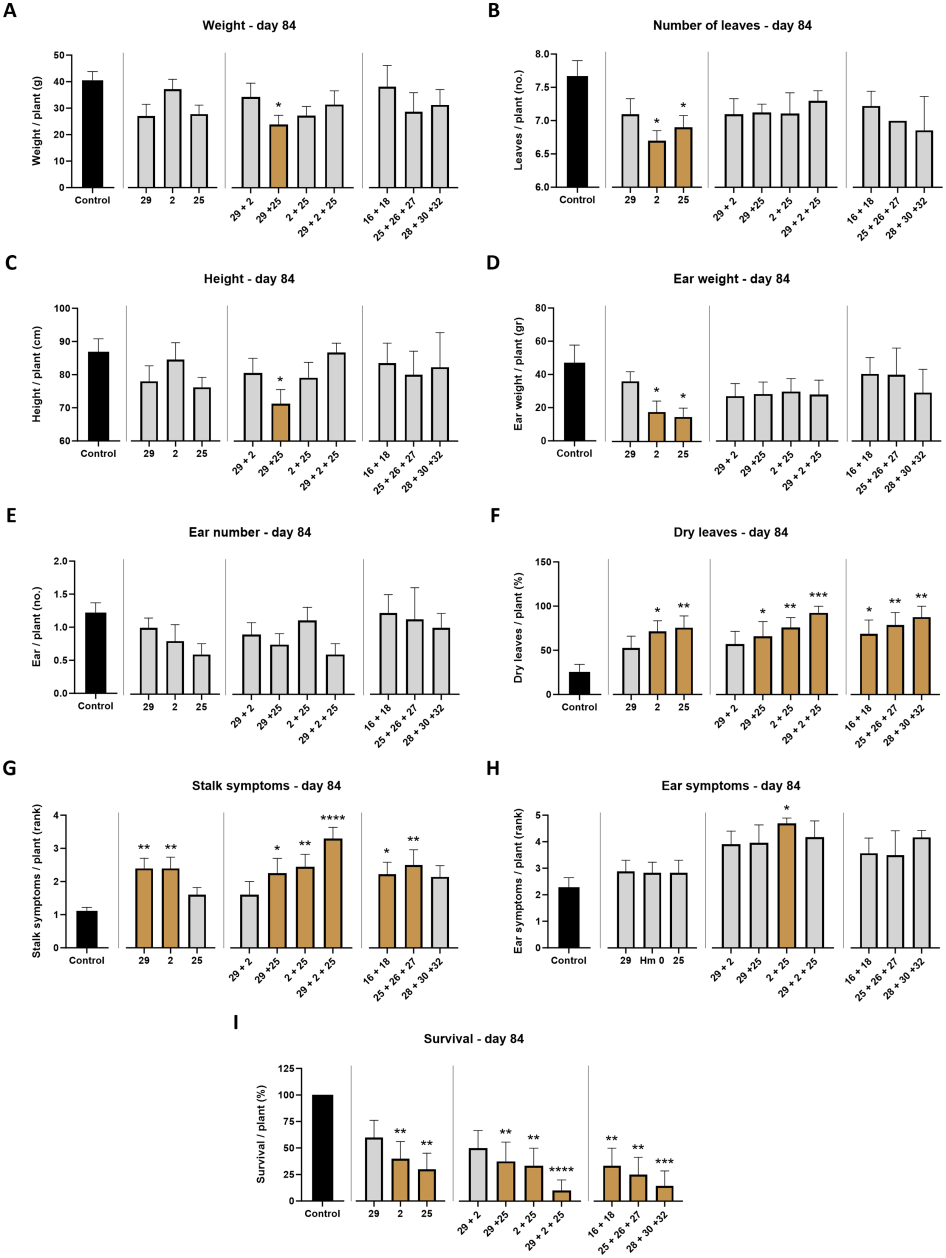
Intra-species interactions impact on mature maize plants (day 84). The effect of *M. maydis* strain mixtures on the development and health of Prelude cv. (late wilt disease susceptible), was studied. The *M. maydis* isolates (Table 1) represent different degrees of aggressiveness in Israel and its regions. These include Mm29 (29, low virulence), Mm2 (2, moderate virulence), and Mm25 (25, high virulence), Beit Keshet Mm16 and Mm18 group (16 + 18, Lower Galilee, northern Israel region), Yavne Mm25, Mm26, and Mm27 group (25 + 26 + 27, southern coastal plain), and Malkia Mm28, Mm30, and Mm32 group (28 + 30 + 32, Upper Galilee, northern Israel area). **(A)** Wet biomass, **(B)** phonological stage (leaves count), **(C)** plant height, **(D)** ear weight, **(E)** ear number, **(F)** number of dry leaves, **(G)** lower stalk symptoms, **(H)** the cobs’ husks symptoms and **(I)** the surviving plants’ rate. Control— healthy, uninfected plants. Each value is a mean of 7–10 repetitions (average plant per treatment). Error bars indicate the standard error. The statistically marked dissimilarity between each group and the control (highlighted in black) was tested using the one-way ANOVA assay and is represented by brown bars and different asterisks above the chart bars (*p < 0.05, **p < 0.005, ***p < 0.0005, ****p < 0.00005).

At harvest (day 84), significant changes between the infected plants and the healthy control plants were recorded (Figure 9). On that sampling day, the combined influence of all strain mixtures (except for Mm29 + Mm2) evoked intense (*p* < 0.05) dry leaves, stalk symptoms, and death but relatively minor growth repression. Most of these strain groups could cause harsher disease symptoms than single strains, even the most virulent Mm25. The results suggest that the pathogenicity of the groups can be ranked from the least to the most aggressive in the following order:

1. Mm16 + Mm18—Beit Keshet, Lower Galilee, northern region, weak pathogenic strains.
2. Mm25, Mm26, and Mm27—Yavne region strains with different LWD aggressiveness.
3. Mm28, Mm30, and Mm 32—Malkia, Upper Galilee, northern region, a combination of high virulence strains.
4. Mm29, Mm2, and Mm25—strains with varied pathogenic traits originated from different regions.

The last group, Mm29, Mm2, and Mm25 (ranked above as the most aggressive among all groups), caused some growth promotion compared to Mm25 alone (without statistical significance) but had a devastating effect on the corn plants’ health. Compared to the non-infected control plants, this subspecies mixture led to higher 262% dry leaves, 46% ear symptoms, 210% stalk symptoms, and 89% death. Regarding the disease signs, this group was more aggressive than Mm25, implying synergistic interactions between the fungal strains. Similarly, the partial combination with Mm2 + Mm25 (the two more virulence strains within this group) evoked a more intense response than Mm25 alone (most observed in 45 and 21% more ear and stalk symptoms).

Regarding the growth symptoms, this last combination (Mm2 + Mm25) led to similar growth as the Mm25 impact, with two exceptions: ear weight (33%) and number (42%) improvement. Therefore, the antagonistic interaction observed on day 42 (Figure 8) between those coupled strains was partly maintained (only in the cobs development) but reversed in the disease indices’ context. The two weak pathogenic stains (Mm29 + Mm2) led, as hypothesized, to a minor LWD, expressed both in relative marginal growth changes and symptoms (with no statistical significance).

**Figure 10:**
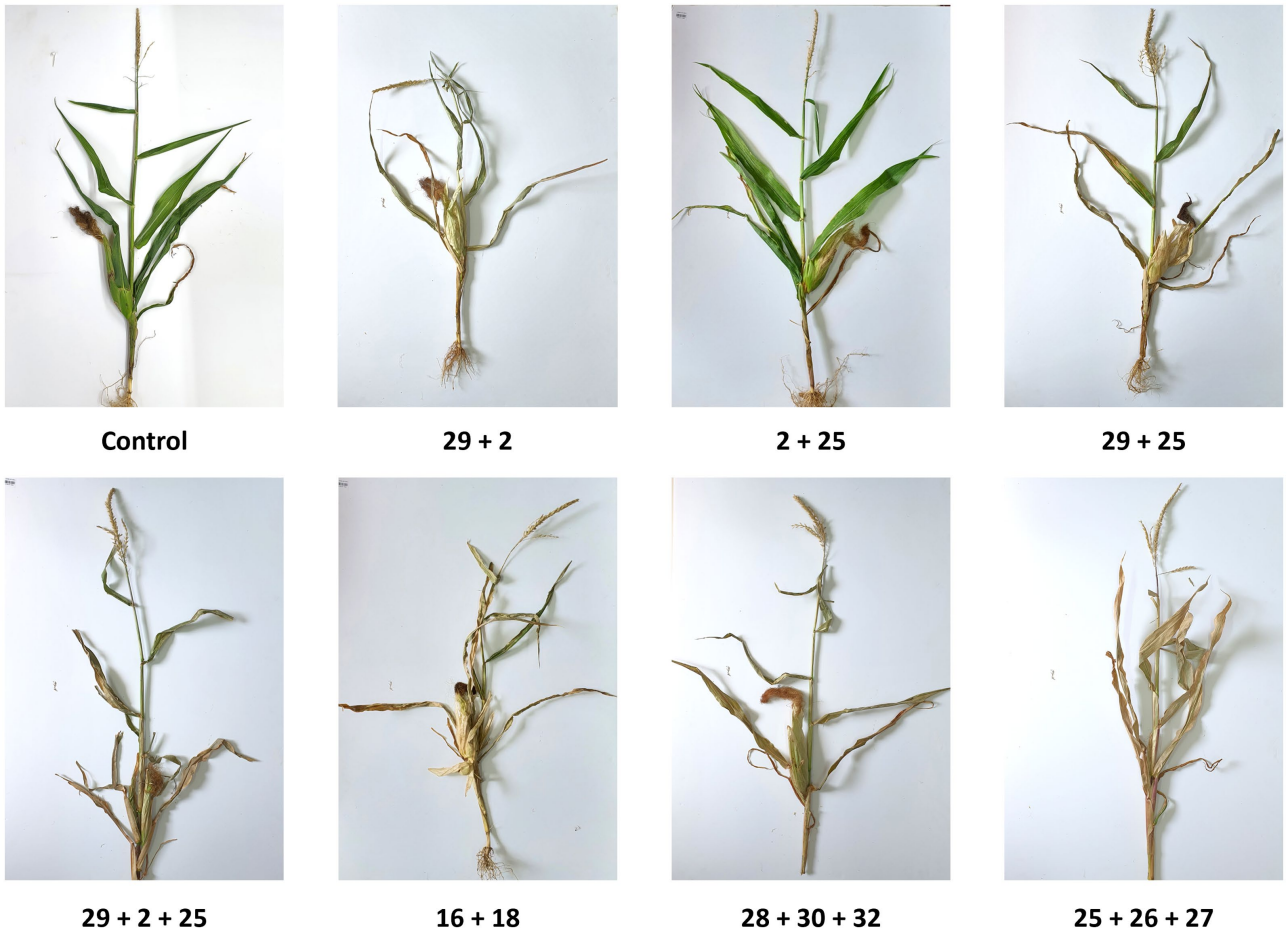
Intra-species interactions impact on day 84—symptoms’ photos of representative plants. The effect of *M. maydis* strain mixtures on the development and health of Prelude cv. (late wilt disease susceptible), was studied. The *M. maydis* isolates (Table 1) represent different degrees of aggressiveness in Israel and its regions. These include Mm29 (29, low virulence), Mm2 (2, moderate virulence), and Mm25 (25, high virulence), Beit Keshet Mm16 and Mm18 group (16 + 18, Lower Galilee, northern Israel region), Yavne Mm25, Mm26, and Mm27 group (25 + 26 + 27, southern coastal plain), and Malkia Mm28, Mm30, and Mm32 group (28 + 30 + 32, Upper Galilee, northern Israel area). A typical plant from each treatment was arbitrarily selected and photographed. In some cases, such as in the 2 + 25 strain combination, there were repeats with mild symptoms and others with severe dehydration. Therefore, selecting one plant that would be a typical sample was impossible.

The photographs of the representative plant samples reflect the above measures well (Figure 10). Still, in some instances, there were variations in LWD symptoms among the plants collected, and it was challenging to choose a fair representative plant (see, for example, Mm2 + Mm25 photo).

**Figure 11:**
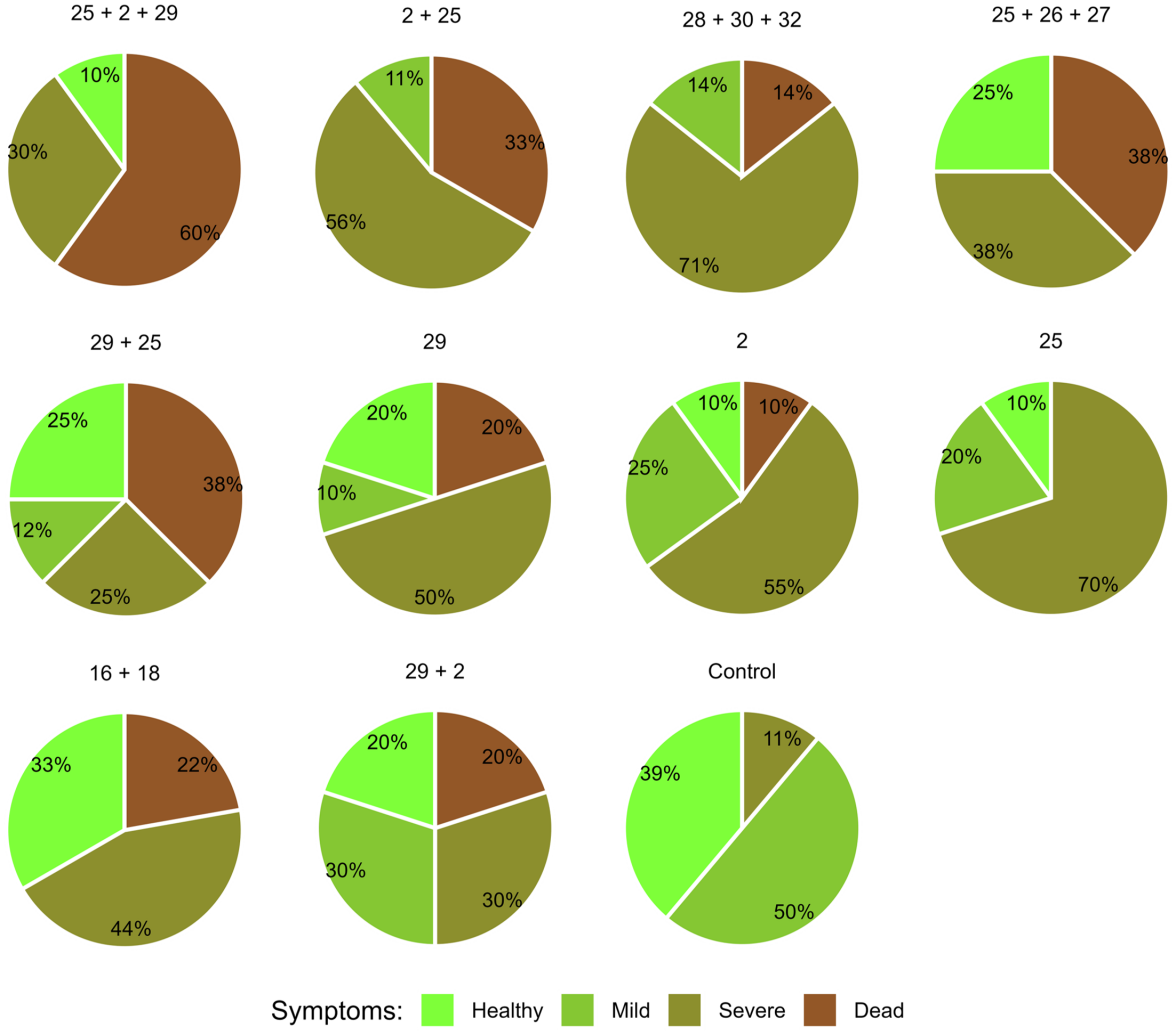
Intra-species interactions impact on day 84—comprehensive wilt disease symptoms evaluation. The effect of *M. maydis* strain mixtures on the development and health of Prelude cv. (late wilt disease susceptible), was studied. The *M. maydis* isolates (Table 1) represent different degrees of aggressiveness in Israel and its regions. These include Mm29 (29, low virulence), Mm2 (2, moderate virulence), and Mm25 (25, high virulence), Beit Keshet Mm16 and Mm18 group (16 + 18, Lower Galilee, northern Israel region), Yavne Mm25, Mm26, and Mm27 group (25 + 26 + 27, southern coastal plain), and Malkia Mm28, Mm30, and Mm32 group (28 + 30 + 32, Upper Galilee, northern Israel area). The overall plant health was estimated on the harvest day using four categories, as described before (Gordani et al., 2023): 1—healthy, 2—mild, 3—severe, and 4—dead.

A comprehensive assessment of plant health at the maturation period (day 84) (Figure 11) corresponds with the wilting signs, discerned through individual examinations of dry leaf count and observable symptoms in lower stems and cobs’ husks. In synthesizing diverse parameters encapsulated within a four-category scale, the mixed region source of strains (Mm29, Mm2, and Mm25) resulted in 60% mortality and only 10% of non-symptomatic healthy plants. The second most damaging infections occurred when the Mm 2 + Mm25 (the more aggressive strains within that group) and Mm28 + Mm 30 + Mm32 (The northern Malkia, high virulence strains) were applied. On the other side of the LWD severity scale are the Mm16 + Mm18 (northern Beit Keshet, weak pathogenic strains) and Mm29 + Mm2 (The northern least pathogenic strains within the mixed origins group). Those combinations had relatively LWD outbreak reflected in 33 and 25% non-symptomatic pants, respectively, and ca. 20% mortality.

Comparative assessment that calculates the total influence of all data gathered in both experimenting days of the semi-field trial (Table 3A) emphasizes the variations in LWD symptoms resulting from the altered fungal strain conjunctions. From this interpretation, it can be observed that in the mixed origins group, applying the three sub-species (Mm29, Mm2, and Mm25) together caused the most severe disease, followed by decreasing symptoms order, according to the strains’ virulence. The other fungal isolate groups were also ranked according to their member aggressiveness, which is in agreement with the results presented above (Figures 8–11).

**Table 3:** Intra-species interactions impact—a comparative evaluation of the growth and survival indices.^a^

(A)															
Treatment	Day 42 (average/pot)						Day 84 (average/plant)							Total (Day 84)	
	Survival (%)	Green leaves (%)	Flowers (no.)	Height (cm)	Leaves (no.)	Weight (g)	Survival (%)	Green leaves (%)	Ear (no.)	Ear weight (g)	Height (cm)	Leaves (no.)	Weight (g)	Ave	Rank
Control	100%	100%	100%	100%	100%	100%	100%	100%	100%	100%	100%	100%	100%	100%	1
29	110%	73%	34%	118%	116%	99%	67%	63%	82%	80%	90%	93%	67%	76%	3
2	86%	75%	80%	94%	88%	98%	33%	38%	65%	39%	97%	87%	92%	67%	6
25	78%	73%	34%	73%	75%	84%	22%	33%	49%	33%	88%	90%	69%	61%	10
29 + 2	90%	56%	57%	89%	95%	88%	56%	58%	74%	59%	93%	93%	85%	75%	4
29 + 25	96%	60%	90%	102%	96%	106%	43%	46%	61%	62%	82%	93%	59%	65%	7
2 + 25	97%	72%	0%	114%	115%	110%	25%	32%	91%	65%	91%	93%	67%	62%	9
29 + 2 + 25	95%	40%	0%	93%	97%	72%	11%	10%	49%	61%	100%	95%	77%	56%	11
16 + 18	78%	86%	57%	74%	81%	80%	38%	42%	100%	88%	96%	94%	94%	78%	2
25 + 26 + 27	114%	47%	71%	130%	131%	119%	25%	29%	92%	87%	92%	91%	71%	70%	5
28 + 30 + 32	94%	77%	46%	88%	96%	71%	17%	16%	82%	64%	95%	89%	77%	63%	8

(B)														
Treatment	Day 42 (average/pot)				Day 84 (average/plant)				Total				All	
	Growth		Symptoms		Growth		Symptoms		Growth		Symptoms			
	Ave	Rank	Ave	Rank	Ave	Rank	Ave	Rank	Ave	Rank	Ave	Rank	Ave	Rank
Control	100%	1	100%	2	100%	1	100%	1	100%	1	100%	1	100%	1
29	92%	2	92%	4	82%	4	68%	3	87%	3	80%	2	82%	3
2	80%	7	90%	5	76%	9	56%	6	83%	6	68%	5	75%	7
25	75%	9	67%	10	66%	11	54%	7	66%	11	65%	8	64%	10
29 + 2	73%	10	82%	7	81%	7	69%	2	81%	8	71%	3	77%	5
29 + 25	78%	8	99%	3	71%	10	58%	5	85%	4	68%	6	76%	6
2 + 25	85%	4	85%	6	81%	5	38%	10	83%	7	61%	10	71%	8
29 + 2 + 25	68%	11	65%	11	77%	8	29%	11	71%	10	48%	11	60%	11
16 + 18	82%	5	73%	9	95%	2	58%	4	84%	5	70%	4	77%	4
25 + 26 + 27	80%	6	113%	1	87%	3	49%	8	100%	2	65%	7	83%	2
28 + 30 + 32	86%	3	75%	8	81%	6	39%	9	78%	9	63%	9	69%	9

a Summary of the open encloser entire season pots trial growth and survival indices results. The effect of *M. maydis* strain mixtures on the development and health of Prelude cv. (late wilt disease susceptible), was studied. The *M. maydis* isolates (Table 1) represent varying aggressiveness across Israel and its regions. These include Mm29 (29, low virulence), Mm2 (2, moderate virulence), and Mm25 (25, high virulence), Beit Keshet Mm16 and Mm18 group (16 + 18, Lower Galilee, northern Israel region), Yavne Mm25, Mm26, and Mm27 group (25 + 26 + 27, southern coastal plain), and Malkia Mm28, Mm30, and Mm32 group (28 + 30 + 32, Upper Galilee, northern Israel area). (A) The results were analyzed by calculating the differences in proportions of each treatment in each sampling day to the control—healthy, non-infected maize plants. The total average for all treatments’ impact on day 84 was calculated, and the treatments were ranked according to their total average (from the highest—number 1 to the lowest—number 11). (B) The growth and health indexes summarization and their total co-influence.

When reviewing the outcomes and the growth and health parameters independently (Table 3B), there is a correspondence between the two measures in most cases. Surprisingly, there were different growth values and disease signs in response to the infection on two occasions. These include the Mm29 + Mm2 strains mixture that severely harmed the growth but minorly manifested in symptoms. In contrast, the Mm25 + Mm26 + Mm27 led to opposite reactions (non-significant growth changes and severe disease symptoms). As in Table 2, the health indexes were more influential at harvest in revealing the disease consequence. Particularly, the greatest impactable value was the survival rate. The most virulent strain composition (Mm29 + Mm2 + Mm25) led to a dramatic mortality increase, from 5% (at mid-season) to 89% (at harvest). Intriguingly, the weak virulent pair, Mm16 + Mm18, showed proportionate high aggression toward maize in sprouts but moderate in mature plants.

**Figure 12:**
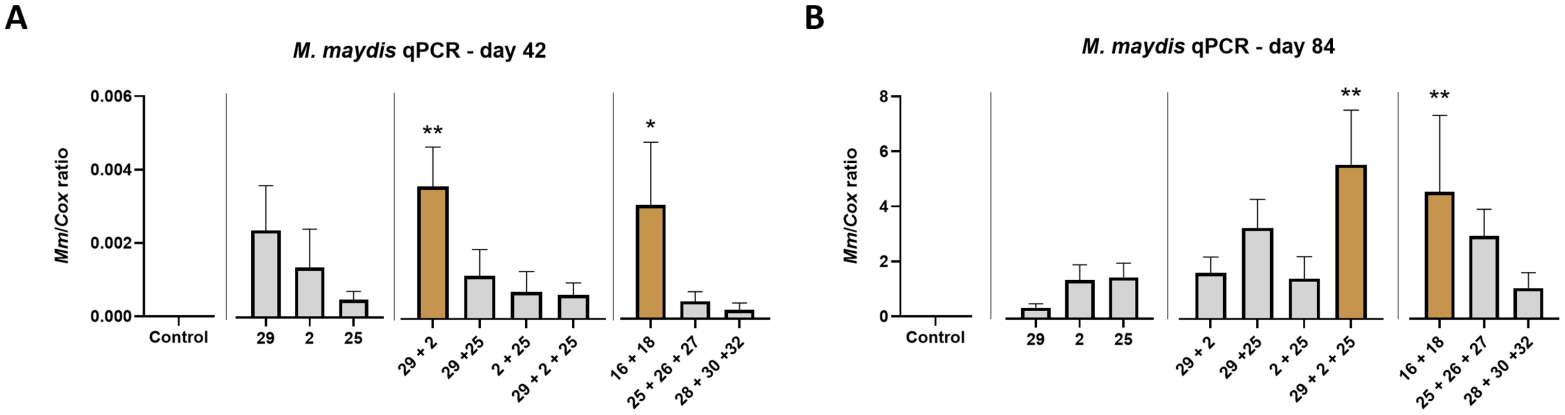
Intra-species interactions impact—Real-Time PCR (qPCR) analysis. The outcome of *M. maydis* virulence on the fungal DNA amount in maize was measured in the roots (day 42, **A**) and the first above soil stalk internode (day 84, **B**). The effect of *M. maydis* strain mixtures on the development and health of Prelude cv. (late wilt disease susceptible), was studied. The *M. maydis* isolates (Table 1) represent varying aggressiveness across Israel and its regions. These include Mm29 (29, low virulence), Mm2 (2, moderate virulence), and Mm25 (25, high virulence), Beit Keshet Mm16 and Mm18 group (16 + 18, Lower Galilee, northern Israel region), Yavne Mm25, Mm26, and Mm27 group (25 + 26 + 27, southern coastal plain), and Malkia Mm28, Mm30, and Mm32 group (28 + 30 + 32, Upper Galilee, northern Israel area). The Y-axis shows the ratio of the specific *M. maydis* DNA fragment to the housekeeping gene-encoding cytochrome C oxidase (COX). Values represent an average of 6–10 repetitions (plants per treatment), except for the 28 + 30 + 32 strains group treatment at day 42, which had three repeats. Error lines represent a standard error. The statistical significance of variance between each group and the control was tested using the one-way ANOVA assay and is emphasized by brown bars and different asterisks above the chart bars (*p < 0.05, **p < 0.005).

Real-Time (qPCR) based analysis conducted on the season mid and end day reveals some similarities and differences in those two growth phases (Figure 12). First, as expected, the pathogen’s DNA in the host tissues peaked during the plant maturation. Second, a more inquiring picture of the impact of diverse strain combinations is revealed. For instance, as in Table 3, the less aggressive couple (Mm16 + Mm 18) had high DNA levels (*p* < 0.05) in the roots of the sprouts (day 42), similar to another relatively reduced virulence couple, Mm29 + Mm2. While the last couple’s DNA maintained low at harvest, the Mm16 + Mm18 group DNA elevated significantly toward the season-ending and resembled the highly aggressive isolates group, Mm29 + Mm2 + Mm25 (the most harmful across all measures present above). Unexpectedly, the highly pathogenic group, Mm28, Mm30, and Mm32, which gained the second aggressiveness high rank in Table 3, had the lowest DNA levels among all groups.

## 4 Discussion

This work examined for the first time the influence of selected Israeli *Magnaporthiopsis maydis* strains, representing varied virulence groups on maize varieties with susceptibility and resistance to late wilt disease (LWD) (Shofman et al., 2022). This strain group was further studied alongside three more isolate groups from different geographical regions across Israel. The maize plants were cultivated in soil infected with those isolates during a whole season in open enclosure pots. This method allowed the best isolation and inspection of the subject matter of interest while mimicking as closely as possible field conditions (Gordani et al., 2023).

Over the years, using resistance maize cultivars to reduce LWD economic impact was a leading strategy in highly affected areas such as Egypt, Israel, and India (Sunitha et al., 2021; Degani et al., 2022b; El-Shenawy et al., 2022; Rakesh et al., 2022; Abdelghany et al., 2023). Such a method gained success and has several advantages, making it a preferable solution over other control methods (Ghazy et al., 2024; Mosa et al., 2023). It is eco-friendly and compatible with any growth method. It is also economically facile but requires constant effort to select and examine new resistant corn cultivars. Continuous cultivation of the same hybrid in infected areas will eventually weaken the plant’s immunity to the disease (Degani et al., 2014; Degani et al., 2019a). The fungus can have pathogenic variations, and this scenario is probably the result of selection pressure that gives an advantage to more virulent strains (Zeller et al., 2002). Thus, studying *M. maydis* communities’ impact on such resistant maize genotypes could provide basic information that may assist in risk assessments and developing new approaches to restrain the disease. The knowledge may also assist in developing rapid corn cultivar resistance assay, as recently reported (Degani et al., 2022b).

### 4.1 The impact of isolates with varied virulence on susceptible and resistant maize cultivars

The first aim of this study was to improve our understanding of the relationships between highly aggressive pathogen strains and resistant corn hybrids. To that end, we selected a highly virulent *M. maydis* strain from our isolate library. This representative isolate and other less aggressive strains were applied to challenged common sweet maize plants, the LWD susceptible Prelude, and the immune Royalty cv. First, as predicted, it was generally observed that the isolates, irrespective of their virulence degree, could harm the sensitive maize cultivar (Prelude) more than the resistant one (Royalty cv.). At the season-ending, the least virulent strain, Mm29, exerted a notable harmful impact on the biomass of the Prelude cv. plants, inducing marked stalk symptoms and a diminished survival rate. That indicates that even such a weak pathogenic strain presents a tangible threat to corn plants susceptible to the disease.

While the Royalty cv. Perform exceptionally well under the disease stress; it did show some weakening when challenged with a highly aggressive strain. In this hybrid, the percentages of dry leaves (the most dominant symptom affected by the fungus at the end of the sprouting phase) drastically drop when the plants mature. Also, the total growth and health indexes (Table 2) recovered with age in Mm2 and Mm25 while remaining high and similar under the less virulent strain (Mm29) impact. This finding reveals some susceptibility in the Royalty cultivar in the juvenile stage (sprouts), from which the plant immunity recovers later. This worrying finding should be explored more in subsequent studies, especially if this temporary weakness can lead to sprout death. Such an alarming situation has already occurred to some extent here, where the most virulent Mm25 isolate caused a 7% fatality rate at mid-season and 11% at harvest in the resistant Royalty cv. plants. Thus, in some environments where such pathogens’ subspecies (or even more virulent strains) are prevalent, the disease can become a real threat to LWD-tolerant cultivars, and other control methods should be considered. Moreover, as proved by the second part of this work (see the next section below), a combination of a few highly virulent strains presents a greater risk, a common situation in commercial fields.

Analyzing the outcomes of the Real-Time PCR (qPCR) analysis associated with the markedly virulent strain (Mm25) unveils a less comprehensible picture (Figure 7). The quantification of DNA levels of this fungal isolate within the Prelude cultivar on the 42nd day of growth and in the Royalty cultivar on the 84th day exhibited relatively low values. This observation lacks a discernible correlation with the manifest disease symptoms, suggesting that heightened virulence may not unequivocally stem from an augmented presence of the fungus within plant tissues. Nevertheless, upon reaching maturity (day 84), there was a notable surge in this pathogen strain’s DNA levels. The amount of DNA in the Prelude cv. increased by 350, 940, and 3,357 times between the two sampling days for the Mm29, Mm2, and Mm25 pathogen isolates, respectively. Thus, the DNA increasing factor, not the highest level, may better indicate the pathogenicity. This assumption needs additional research for better verification.

It seems reasonable that the pathogen infection disrupted the flowering at the end of the sprouting phase (day 42). This phenomenon is probably related to the development delay caused by the fungus. Examining this response in both trials suggests a correlation between the pathogen aggressiveness and the flowering delay in the susceptible Prelude cv. However, in the LWD tolerance Royalty cv., an unexpectedly relatively higher flowering was measured in the most virulent strain (Mm25, Figure 3D).

One elucidation posits that this adaptive mechanism enhances the perpetuation of the species’ survival in suboptimal environmental conditions (Wada and Takeno, 2010). Indeed, plants can adjust their developmental progress in reaction to various stressors. In the context of pathogen-induced stress, plants may instigate the onset of flowering as an expedient reproductive strategy to secure the production of offspring (Takeno, 2016). As far as we know, this is the first report on enhanced development response to *M. maydis*’s induced stress. Exploring this behavior further and revealing its triggers and mechanisms will be most interesting, especially since the reaction was temporary and did not affect the season-ending disease outburst.

We can get insights about the phenomenon by reviewing the literature. The root-infecting fungal pathogen *Fusarium oxysporum* accelerates flowering in Arabidopsis (Korves and Bergelson, 2003; Lyons et al., 2015). Pathogen infection generally increases salicylic acid, jasmonic acid, and ethylene levels, suggesting a possible involvement of these hormones in flowering accelerated by pathogen infection (Korves and Bergelson, 2003). While the plant immunity may have protected it from the weaker *M. maydis* strains, the inoculation pressure posed by the riskier isolate could have overcome the plant’s tolerance and evoked flowers’ growth acceleration.

### 4.2 The impact of *M. maydis* isolates’ origin and intra-species interactions on late wilt disease

The following research effort of this work was dedicated to *M. maydis* isolates groups to elucidate fungal strain–strain cross-talk and co-influence on the disease progression. At mid-season sampling (day 42) across various metrics (except for the number of dry leaves), it is evident that dual infecting the plants with the two more aggressive isolates (Mm2 and Mm 25) resulted in attenuated disease manifestation compared to Mm25, as evidenced by elevated growth parameters and reduced disease symptoms. These findings imply antagonistic interactions between the above sub-species. To support this, the mixed-region source of strains (Mm29, Mm2, and Mm25) group exhibited even greater antagonism (as measured by growth indexes) than Mm25 alone. This result might have gained more statistical power to reveal significant differences if the number of repetitions had not been reduced to three due to this group’s high mortality of sprouts. Therefore, it is possible that this strain combination influences disease but has a less pronounced effect on growth reduction in that plant age (see Table 3).

On the same growth day (day 42), the Mm25, Mm26, and Mm27 (strains with diverse virulence degrees from the Yavne, southern coastal plain) showed similar and even intense antagonism regarding growth and survival measurements compared to the Mm25 treatment. The third set of strains denoted as Mm28, Mm30, and Mm32, which are representative of the Malkia, Upper Galilee, northern Israel area, was anticipated to induce a pronounced outbreak of LWD due to the inclusion of strains characterized by comparatively elevated virulence. Instead, plants infected with these strains had, in most measures, improved growth and survival rates. An exception was the weak virulence group from Beit Keshet (Mm16 + Mm18), which showed synergy at the end of the sprouting phase in all measures (with growth suppression, almost severe as Mm25).

At harvest (day 84), this image was reversed. The co-inoculation involving Mm2 and Mm25 elicited a heightened host response (growth reduction with few exceptions and increased ear and stalk symptoms) compared to the infection with Mm25 alone. Consequently, the antagonistic interplay witnessed on the 42nd day among the two strains persisted partially, specifically in the maturation of cobs, while exhibiting a reversal in the context of disease indices. Moreover, adding the third weak pathogenic strain, Mm29, to this couple (Mm2 and Mm25) led to more severe disease, indicating stronger synergism in evoking LWD. Thus, it may be concluded that antagonism and synergism exist within *M. maydis* populations, mainly evident at the plant sprouting phase. Is the most intense competition more prevalent within highly aggressive strain communities? The above-presented evidence, an antagonism in sprouts, and the full destructive potential revealed toward the harvest support such thinking. Yet, those new and few cases gained here should open the door for a deeper investigation.

Unexpectedly, disparate growth metrics and disease manifestations were observed in reaction to the infection in two distinct instances. Notably, the combination of Mm29 and Mm2 strains resulted in substantial impediments to growth with relatively modest symptomatic expressions. Conversely, the assemblage of Mm25, Mm26, and Mm27 yielded inverse outcomes, characterized by negligible alterations in growth parameters alongside pronounced and severe disease symptoms. It may be inferred from these results that some subspecies groups specialize in growth (phenological development and above-ground parts weight and height) disruption and others in wilting evoking that may be attributed to their biotrophic or necrotrophic nature. Still, the picture is more complex since the plant responses are age-dependent.

In both trials, the healthiness parameters were more influential at harvest in exposing the LWD outcome. Specifically, the primary impactable measure was the survival value. The most pathogenic isolates mixture (Mm29 + Mm2 + Mm25) infection resulted in a drastic death increase (84%) toward the harvest. Curiously, the weakly aggressive couple, Mm16 + Mm18, could induce relatively high disease in sprouts but moderate in mature plants. The above examples imply that the *M. maydis* communities’ ability to generate illness depends on the plant’s age-related immunity, as suggested earlier (Sabet et al., 1970b).

The DNA levels of the Mm16 + Mm18 group exhibited a marked increase as the season approached its conclusion, resembling the DNA profile of the isolates group Mm29 + Mm2 + Mm25, which demonstrated the highest degree of virulence across all metrics studied. Contrarily, the highly pathogenic group comprising Mm28, Mm30, and Mm32, although attaining the second-highest rank in aggressiveness (Table 3), displayed the lowest DNA levels among all experimental groups, presenting an unexpected outcome. So, as in the first trial, there seems to be no clear correlation between DNA levels and aggressiveness. However, such a correlation appeared if we examined the DNA changes between the two sampling days. In the case of the Mm28, Mm30, and Mm32 strains’ group, the pathogens’ DNA levels in the plant peaked by a factor of 4,950. This DNA elevation was the second most pronounced among all groups tested. Since some groups had a low starting point result from low infectivity in sprouts (for example, the Mm25, Mm26, and Mm27 group that gained the highest DNA increase, 6,159-fold), both the final fungal DNA levels and its increasing factor should take into account to better evaluate the strains’ pathogenicity level.

### 4.3 Deeper understanding of the interactions between the pathogen’s strains

Most plants can host a variety of pathogen species or different strains of the same species (Degani et al., 2024a,b). While interactions between different fungal pathogens have been studied intensively, the intraspecies cross-talk and its impact on the host’s health is less known. Antagonistic interactions may occur through competition for space or nutrients, plant susceptibility changes due to induced resistance, or metabolite production by one pathogen strain that suppresses the other. On the other hand, subspecies that are less competitive with each other can lead to enhanced diseases. They can interact synergistically through various mechanisms, including chemical signaling that affects gene expression and metabolic exchange or complementarity that reduces competition for nutrients and enhances the metabolic capabilities of the group (Frey-Klett et al., 2011). As demonstrated here for Mm25, Mm26, and Mm27 (strains with varied virulence degrees collected from the same location), strains specializing in biotrophic lifestyles may coexist with more necrotrophic strains, resulting in more severe disease. The sequence in which a host is infected by each pathogen in a complex disease, along with the pathogen’s trophic level, may influence the type of interaction these pathogens ultimately establish during the progression of the disease. Still, our understanding of the mechanisms driving geographic variation, the prevalence of specific pathogen strains in plants, and their effect on complex diseases is quite limited (Lamichhane and Venturi, 2015). Uncovering the underlying mechanisms behind the subspecies of crosstalk dynamics, including their interactions with the plant, requires in-depth future research.

*Magnaporthiopsis maydis*’s diversity of aggression was previously reported in the Iberian Peninsula (García-Carneros et al., 2011; Ortiz-Bustos et al., 2015) and Egypt (Zeller et al., 2002). Most works focused on testing single isolates against susceptible and resistant maize hybrids. Only Zeller et al. (2002) researched the competitive ability in multiple infections. Still, they scored only the absence or presence of disease and could not determine if there were phenotypic variations between single and multiple infections. In a highly virulent strain, they found an apparent negative correlation between sole infection and multiple strains inoculation (i.e., drastic reduction in the pathogen prevalence). This finding aligns with the current work’s conclusion regarding *M. maydis* intra-species antagonistic interactions.

Yet, many other factors can influence those strain–strain interactions. Variations in growth capabilities, whether in soil or the host plants, can be attributed to physical environment properties or biotic interactions. For example, the differences in sensitivity to other soil or plant microbes or their metabolic byproducts can be crucial (El-Assiuty et al., 1991; Ghazy and El-Nahrawy, 2020; Degani et al., 2021a; Degani and Gordani, 2022). Soil inoculation density is a pivotal factor influencing disease severity (Yassin and El-Naggar, 2024). Additionally, variances may exist in the *M. maydis* sub-species capacity to produce cellulases and other degradative enzymes necessary for penetrating plant roots and facilitating colonization.

The competition within *M. maydis* communities may lead to selection pressure, resulting in changes in the pathogen nature. Some species may favor a more biotrophic lifestyle to avoid competition, while others may prefer a necrotrophic adaptation. The data presented here and earlier (Shofman et al., 2022) support such a possibility. Zeller et al. (2002) identified generally weakened *M. maydis* strains in three of the four clonal lineages tested but have not identified any standard features that these reduced virulence lineages share, and the researchers stated that this low pathogenicity could be due to multiple causes. Therefore, answering this question will require further work to resolve.

Today, the information regarding the combined influence of several *M. maydis* isolates is scarce. Additional investigations should delve into the molecular composition of fungal populations in highly affected areas such as the Iberian Peninsula, Egypt, India, and Israel. Furthermore, conducting a global study on *M. maydis* in regions with reported cases will help determine if the lineages present in Israel are the same or different from those identified in other countries. Additionally, this study could shed light on whether the aggressiveness of *M. maydis* from the Iberian Peninsula and Egypt varies compared to that of Israel’s pathogen population.

It will be most interesting to expand the research and examine additional *M. maydis* communities. Different compositions of the pathogen isolates can contribute to a better understanding of the population and its damage potential. The geographic distribution of *M. maydis*, as identified in this study, may be attributed to late wilt severity. An accurate understanding of the aggressiveness of *M. maydis* is crucial for pinpointing effective sources of resistance to the disease. Such works can take advantage of the soil bioassay developed earlier (Degani et al., 2020b) since isolating, identifying, and studying more *M. maydis* strains from a diverse geographic location is essential to accurately understand the pathogen population, its local communities, and the risk they pose.

Finally, it should be considered that *M. maydis* strains are not alone in the soil and the plant environment. Other pathogens’ presence, interactions, and the stresses they cause on the plant host can completely change the disease outcome (Drori et al., 2013; Farahat et al., 2020; Degani et al., 2022a). Interactions between different species can be notably intricate, particularly due to their dependence on the surrounding conditions, encompassing both host and environment, which can significantly influence their activities (Deveau et al., 2018; Darwesh and Elshahawy, 2023). The complexity is further heightened when considering the soil microbiome and its impact on the survival capabilities of individual pathogens in the exposed terrain, particularly over extended durations. In conjunction with the soil microbiome, protective endophytes residing within the plants serve as an additional defense mechanism (Gond et al., 2015; Degani et al., 2021a; Lin et al., 2022), thereby emphasizing the indispensability of both elements for prospective scientific investigations.

## 5 Conclusion

Maize late wilt disease (LWD), caused by the phytopathogenic fungus *Magnaporthiopsis maydis*, poses significant challenges in endemic regions such as Israel, Egypt, Spain, Portugal, and India. This study provides valuable insights into the pathogen’s behavior and interactions that can lead to better management of LWD. The main findings revealed the risks of highly aggressive fungal isolates, antagonistic and synergistic interactions among fungal strains and their season age-related influence, and the diverse pathogenic effects based on fungal subspecies’ biotrophic or necrotrophic nature. Developing maize varieties with multi-layered resistance mechanisms targeting different fungal subspecies is crucial. Specifically, genomic tools should be employed to identify resistance traits that are less likely to be overcome by evolving aggressive fungal strains, ensuring the durability of resistance in high-risk regions. We should design tools to monitor and predict pathogen dynamics, incorporating stress-induced flowering as an early indicator of infection severity. Additionally, soil and seed testing, alongside diagnostic tools for identifying specific fungal strains or mixtures, can improve our risk assessment accuracy and targeted management based on local pathogen profiles. These may include harnessing friendly microorganisms that enhance the suppressive effects between fungal strains during early crop stages. Integrated management practices, such as crop rotation, soil modifications, and precision agriculture tools, can further disrupt the ecological balance favoring aggressive strains. Additionally, targeted chemicals or biofungicide applications tailored to inhibit specific pathogenic behaviors may help mitigate the disease’s effects. By applying these strategies, the study’s findings can contribute significantly to controlling LWD and reducing its impact in affected regions.

## Author’s note

The current work is part of a continuous scientific effort to advance our understanding of maize late wilt disease (LWD) and its causative agent, the fungus *Magnaporthiopsis maydis*. The primary management method involves cultivating resistant maize varieties, but it faces challenges as the plants' immunity compromises over time. Moreover, intra-species interactions and their co-influence on disease severity are poorly understood. The current study revealed complex interactions in mixed fungal strain populations, emphasizing the destructive potential of some strains on resistant cultivars and their potential economic impact. Additionally, subspecies' specialization in growth disruption or wilting suggests their biotrophic or necrotrophic nature. The research results contribute to understanding LWD, the pathogen communities, and their host-plant interactions. The revelation of maize age-related immunity's role in LWD generation adds depth to comprehending the associated pathogen's role. Such new data are essential for risk assessment and for developing new control strategies to mitigate the disease.

## Data Availability

The original contributions presented in the study are included in the article/Supplementary material, further inquiries can be directed to the corresponding author.
