# Supplementary material for: Mixed fungal strains challenge host resistance: insights into Magnaporthiopsis maydis pathogenicity in maize
Source: Front Microbiol. 2025 Jan 27;16:1520237. doi: 10.3389/fmicb.2025.1520237 (PMC11808155; doi:10.3389/fmicb.2025.1520237)
Supplement: Supplementary file 1 [file Table_1.DOCX]

**Table S1.** Comparison of the Prelude and Royalty sweet maize cultivars' characteristics

| **Prelude cv.** | **Royalty cv.** | **Cultivar** |
| --- | --- | --- |
| SRS Snowy River Seeds, Australia | Pop Vriend Seeds B.V., Andijk, The Netherlands | Producer |
| Green 2000 Ltd., Israel | Eden Seeds, Reut, Israel | Supplier |
| low | high | Late wilt disease resistance |
| 50-57 ^a, c^ | 50-55 ^c, d^ | Days to 70% silk |
| 7 ^a^ | 6-7 ^b, d^ | Plants/m^2^ |
| 200 ^e^ | 162-200 ^d, i^ | Plant height (cm) |
| 80 ^e^ | 67.8-82.8 ^d, i^ | Height to the cob base (cm) |
| 2.2-2.5 ^a^ | 2.2-2.6 ^b, d^ | Yield in healthy fields (kg/m^2^) |
| 18-20 ^e, f.^ | 16-18 ^d, e, i, k^ | Number of rows in the cob |
| 18.9-20 ^e, f^ | 16.7-21 ^d, e, i, k^ | Cob length (cm) |
| 5.3-5.4 ^f^ | 4.8-5.2 ^d, i^ | Cob width (cm) |

^a^ Degani et al. (2018) (Degani et al., 2018). ^b^ Drori et al. (2013) (Drori et al., 2013). ^c^ Degani et al. (2019) (Degani et al., 2019a). ^d^ Sweet corn variety field test, Eden experimental Farm (North Israel R&D, Israel Ministry of Agriculture), spring 2016 (Mivzak Yerakot - field and vegetable, No. 303, Vegetable Growers Organization journal, April 2017, pp 35-38). ^e^ The supplier, GadotAgro product website ([link](https://www.gadotagro.com/products/%D7%AA%D7%99%D7%A8%D7%A1-%D7%9E%D7%AA%D7%95%D7%A7-su/)). ^f^ The manufacturer SNOWY RIVER breeds website ([link](https://www.snowyriverseeds.com/varieties/sweet-corn/prelude)). ^k^ The manufacturer Pop Vriend Royalty cv. datasheet ([link](chrome-extension://efaidnbmnnnibpcajpcglclefindmkaj/https:/seedplus.hu/wp-content/uploads/2017/11/pvs-csemegekukorica-vetomag-katalogus-2017.pdf)). ^i^ Sweet corn variety field test, Hulata (North Israel R&D, Israel Ministry of Agriculture report), Summer 2005.
